# Aberrant Dynamic Connectivity for Fear Processing in Anorexia Nervosa and Body Dysmorphic Disorder

**DOI:** 10.3389/fpsyt.2018.00273

**Published:** 2018-06-26

**Authors:** D. Rangaprakash, Cara Bohon, Katherine E. Lawrence, Teena Moody, Francesca Morfini, Sahib S. Khalsa, Michael Strober, Jamie D. Feusner

**Affiliations:** ^1^Department of Psychiatry and Biobehavioral Sciences, University of California, Los Angeles, Los Angeles, CA, United States; ^2^Department of Psychiatry and Behavioral Sciences, Stanford University, Stanford, CA, United States; ^3^Oxley College of Health Sciences, University of Tulsa, Tulsa, OK, United States; ^4^Laureate Institute for Brain Research, University of Tulsa, Tulsa, OK, United States

**Keywords:** fearful face processing, dynamic effective connectivity, fronto-limbic modulation, anorexia nervosa, body dysmorphic disorder

## Abstract

Anorexia nervosa (AN) and body dysmorphic disorder (BDD) share distorted perceptions of appearance with extreme negative emotion, yet the neural phenotypes of emotion processing remain underexplored in them, and they have never been directly compared. We sought to determine if shared and disorder-specific fronto-limbic connectivity patterns characterize these disorders. FMRI data was obtained from three unmedicated groups: BDD (*n* = 32), weight-restored AN (*n* = 25), and healthy controls (HC; *n* = 37), while they viewed fearful faces and rated their own degree of fearfulness in response. We performed dynamic effective connectivity modeling with medial prefrontal cortex (mPFC), rostral anterior cingulate cortex (rACC), and amygdala as regions-of-interest (ROI), and assessed associations between connectivity and clinical variables. HCs exhibited significant within-group bidirectional mPFC-amygdala connectivity, which increased across the blocks, whereas BDD participants exhibited only significant mPFC-to-amygdala connectivity (*P* < 0.05, family-wise error corrected). In contrast, participants with AN lacked significant prefrontal-amygdala connectivity in either direction. AN showed significantly weaker mPFC-to-amygdala connectivity compared to HCs (*P* = 0.0015) and BDD (*P* = 0.0050). The mPFC-to-amygdala connectivity was associated with greater subjective fear ratings (*R*^2^ = 0.11, *P* = 0.0016), eating disorder symptoms (*R*^2^ = 0.33, *P* = 0.0029), and anxiety (*R*^2^ = 0.29, *P* = 0.0055) intensity scores. Our findings, which suggest a complex nosological relationship, have implications for understanding emotion regulation circuitry in these related psychiatric disorders, and may have relevance for current and novel therapeutic approaches.

## Introduction

Body dysmorphic disorder (BDD) and anorexia nervosa (AN) are psychiatric disorders with prevalences of ~2% (BDD) (1) and 1% (AN, in females) (2) in the general population. Both disorders have a cardinal identifying feature in common: a dramatically distorted, anxiety-ridden perception of appearance. BDD and eating disorders commonly co-occur within individuals; ~32% of individuals with BDD have a lifetime eating disorder and 25–39% of those with AN have, or have had, BDD (3, 4). However, they are deemed to be nosologically independent (5). There are differences between the two— in BDD the sex distribution is roughly equal, in contrast to strong female preponderance in AN (6), and the preoccupations associated with BDD typically focus on imagined defects on the face and head (7), whereas AN is distinguished by a phobic avoidance of normative body mass even when confronted with extreme malnutrition. Nevertheless, there is considerable overlap in appearance concerns for specific body parts such as the size of abdomen, hips, and thighs (8). Approximately 30% of those with BDD have weight-related concerns (9). Moreover, we recently found that those with AN and those with BDD show similar subjective ratings of others' bodies as being more overweight, others' face images (high and low detail) as being less attractive, and were more likely to be triggered to think of their own appearance when viewing faces and bodies, compared with healthy controls (10).

In addition, recent evidence from our laboratory shows that the syndromes display similar brain activation abnormalities in visual systems when viewing faces and houses (11), supporting the idea that they might represent variants of a shared body image diathesis (12). Emerging evidence of genetic correlations across psychiatric phenotypes, long considered qualitatively separate, lend support to the notion (13). Relatedly, a small twin study of males with AN found a high proportion of their co-twins to have BDD symptoms (14). However, the question of whether or not common and disorder-unique neural phenotypes characterize the relationship between BDD and AN remains unstudied.

The observation that anxious traits emerge early in AN and are familially transmitted (15), lends credence to the possibility that cortico-limbic dysfunction implicated in anxiety might also be a mechanistic process in AN. Emerging evidence suggests emotion regulation and social cognition difficulties in AN (16, 17). A study in underweight patients using electroencephalography (EEG), showing reduced visual evoked P300 potentials in response to negatively valenced faces (18) as well as a functional magnetic resonance imaging (fMRI) study showing abnormally low amygdala activation to fearful faces (19) lend support. By contrast, the only comparable study in BDD that we are aware of showed an absence of limbic hyperactivity when participants viewed their own faces, despite high subjective rating of emotional discomfort (20). Analogously, we note here that some fMRI studies in obsessive-compulsive disorder (OCD), a condition closely related to BDD, have similarly shown an absence of amygdala hyperactivity upon exposure to negatively valenced stimuli (21–27) [although see (28–30)].

Abnormal fronto-limbic connectivity has been associated with anxious traits (31). Fronto-limbic effective connectivity can be considered as a top-down process, wherein prefrontal regions exercise (mostly) inhibitory control over limbic regions like amygdala, thus reducing limbic hyperactivity (32). As such, an increasing influence of prefrontal regions over limbic regions over time should manifest as increasing fronto-limbic connectivity over time. An impairment of such a mechanism, observed through lack of increasing fronto-limbic connectivity over time, would then be indicative of impaired top-down regulation. In this study, we probed such a temporal change in fronto-limbic effective connectivity in AN as compared to BDD and healthy controls (HC).

The neural response to fearful faces is a well-established laboratory technique for interrogating fronto-limbic modulation of emotion-regulating circuitry (33). The activating property of the exposure is robust, recruiting limbic regions including, but not limited to, the amygdala and the medial prefrontal cortex (mPFC) (33); notably, the right-amygdala appears to mediate fear processing to a greater degree than the left-amygdala (34). Also well-established is the modulation of amygdala excitement by the mPFC when negatively valenced stimuli are presented repeatedly in the absence of threat or reward (35, 36).

With this as background, we present what is to our knowledge the first study to examine, and compare, fronto-limbic connectivity in these two body image phenotypes. Specifically, we probed fronto-limbic connections evoked by self-labeling of emotional responses (37, 38), and compared directional connectivity between bilateral medial-prefrontal cortex (mPFC), rostral anterior-cingulate cortex (rACC) and amygdala in BDD, AN, and healthy controls, based on previous research focused on the engagement of these regions by fearful faces (33). We previously compared functional connectivity when viewing neutral-expression faces in lower- and higher-order visual processing systems in BDD, AN, and healthy controls (39). Another study, in recovered AN participants, probed activation when participants labeled the gender while viewing fearful faces (40). Yet, no studies have compared connectivity associated with viewing emotional faces in these populations.

In this study, we employed dynamic effective connectivity modeling (41) to estimate and assess the change in connectivity over time with repeated exposure to fearful faces. Given that a temporal reduction of limbic response to arousing stimuli during fear processing has previously been found (37), we probed the within-group change in connectivity across fearful-face task blocks, in addition to comparing between-group connectivity differences. We hypothesized that: (1) healthy controls would exhibit significantly increasing bidirectional fronto-limbic connectivity over time with repeated stimulus exposure; (2) given the evidence that cognitive behavioral therapy (CBT) techniques, utilizing exposures, appears to be at least moderately beneficial in BDD (42, 43), we predicted significantly increasing prefrontal-to-amygdala connectivity, but given the evidence of limbic hypo-responsiveness in BDD (20), we predicted no significant increases in amygdala-to-prefrontal connectivity in BDD; and (3) based on strong evidence of premorbid anxious traits (15) and familial transmission of anxiety (13) in AN, and the generally poor to modest response to traditional CBT approaches that utilize exposure techniques (44), we predicted no significant increases in bidirectional fronto-limbic connectivity in AN. Finally, we hypothesized that fronto-limbic connectivity would be associated with anxiety symptoms across AN and BDD, and with core AN and BDD symptoms.

## Methods

### Participants

We recruited unmedicated young individuals (aged 14–38 years) in three groups of equivalent age, sex, and education: BDD (*n* = 32); weight-restored AN (*n* = 25); and HC (*n* = 37). The study was approved by the UCLA Institutional Review Board. Informed written consent was obtained from all participants. Please refer to Supplemental Information SI-1.1 for further information.

Participants were right-handed as determined by the Edinburgh handedness test (45) with normal, or corrected, visual acuity as tested with a Snellen eye chart. AN participants were required to have a body mass index (BMI) ≥ 18.5, but at the time of the study to have met all other DSM-IV criteria for the illness (except for amenorrhea). We recruited weight-restored participants to avoid the likely confounding effects of starvation on brain activity. Clinical evaluation of the BDD sample was performed by JDF, and the AN sample by CB, MS, and SSK.

#### Inclusion/exclusion criteria

To assure that AN and BDD participants were representative of general clinical populations, we allowed current DSM-IV diagnoses of dysthymia, OCD, major depression, panic disorder, generalized anxiety disorder, social phobia, or agoraphobia. Controls were excluded if they had lifetime bipolar disorder or psychosis, and any current DSM-IV Axis I disorder. For the AN group, a current or lifetime history of BDD was exclusionary, as was AN for the in the BDD group. Other exclusionary criteria, applied to all participants, included suicidality, self-injurious behavior, lifetime neurological disorder, current pregnancy, any medical illness that could affect cerebral metabolism, or current treatment with cognitive behavior therapy. Participants were required to be free from psychoactive medications for at least 8 weeks prior to entering the study. Nine AN and 1 BDD participant were under outpatient psychotherapy treatment at the time of the study. Twenty-four AN participants met DSM-IV criteria for restricting type; while, one had binge eating/purging type, and was included in the final analysis after confirming that this person's connectivity findings were not an outlier. None of the participants had amenorrhea.

#### Screening/clinical measures

Participants were administered the Mini International Neuropsychiatric Interview (MINI) (46), and the BDD Diagnostic Module (47) modeled after the DSM-IV. Severity of psychiatric symptoms was quantified using the Hamilton Anxiety Rating Scale (HAMA) (48), Brown Assessment of Beliefs Scale (BABS) (49), and Montgomery-Åsberg Depression Rating Scale (MADRS) (50). BDD participants received the BDD version of the Yale-Brown Obsessive Compulsive Scale (BDD-YBOCS) (51), and AN participants received a modified version of the Eating Disorder Evaluation (EDE) Edition 16.0D (52), and the Yale-Brown-Cornell (YBC) eating disorder scale (53).

### Imaging data acquisition

Functional MRI data were acquired on a Siemens Trio 3T scanner using a T2^*^-weighted echo planar imaging (EPI) gradient-echo pulse sequence (repetition time/echo time = 2,500/25 ms; flip angle = 80°; matrix = 64 × 64; field-of-view = 192 mm; in-plane voxel-size = 3 mm^3^; 0.75 mm intervening spaces; 32 total interleaved slices). Higher resolution matched-bandwidth (MBW) T2 and T1-weighted images were collected for coregistration.

### Fearful face task

Fearful face stimuli were presented in a blocked design with 4 unique stimuli per block for three blocks, to assess change in connectivity across the three blocks. Each face stimuli appeared for 4 s with an inter-stimulus interval of 500 ms, and each block was 18 s long. There were also blocks of scrambled faces of the same duration and with the same inter-stimulus intervals. The fearful face and scrambled face blocks alternated, so that the entire run was comprised as follows: fearful face block 1, scrambled face block 1, fearful face block 2, scrambled face block 2, fearful face block 3, and scrambled face block 3. Participants indicated level of fearfulness when viewing faces (subjective fear rating [SFR]) by pressing buttons 1 (minimum) through 4 (maximum), or to indicate the roundness of a scrambled face (as the control task) by pressing button 1 (circle) or 2 (oval). We asked participants to rate their own emotional state during fearful face viewing (vs. rating the degree of fear expressed by the face), in order to engage top-down modulation of emotion processing. For further details, please refer to Supplemental Information SI-1.2. In addition to assessing temporal change in connectivity in this study, we also performed an omnibus ANOVA on SFR with group and fearful face block as factors, followed by an assessment of the within-group change in SFR across the three fearful face blocks and between-group SFR differences (ANOVA followed by *T*-tests in both cases, *P* < 0.05, Bonferroni corrected).

### FMRI data preprocessing

**Figure 1:**
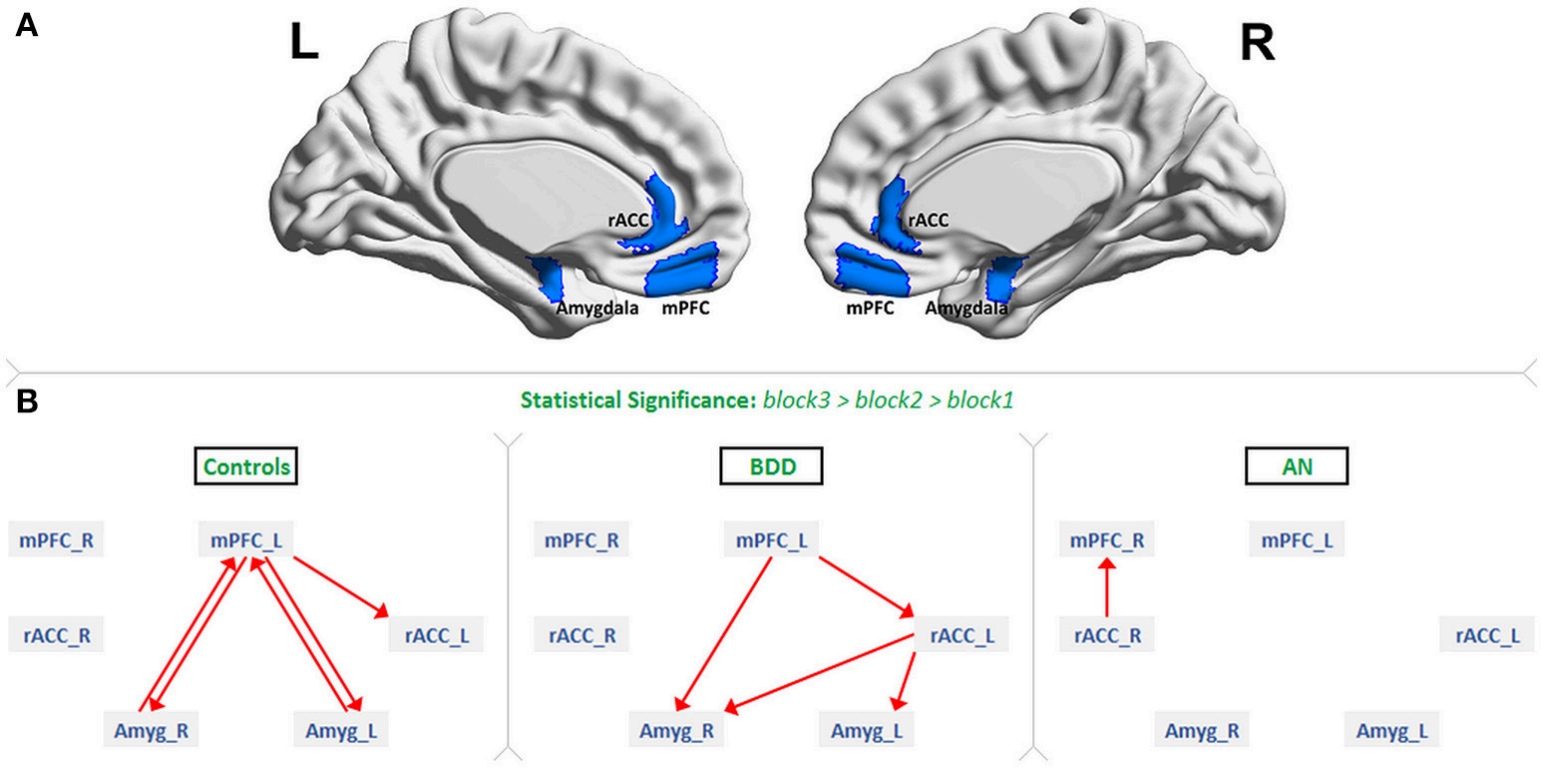
The regions of interest and dynamic effective connectivity findings: **(A)** Regions-of-interest: bilateral medial prefrontal cortex (mPFC), rostral anterior cingulate cortex (rACC) and amygdala. **(B)** Significant connections identified in each of the three groups as increasing from block1 to block2 to block3 during the presentation of fearful face stimuli. This figure shows the evidence we found for all our three hypotheses. As predicted, there was bidirectional connectivity between mPFC and amygdala in HCs, unidirectional mPFC-to-amygdala connectivity in BDD, and no significant prefrontal-amygdala connectivity in AN.

FMRI data were preprocessed in the FMRIB Software Library (FSL version 5.0.6), including motion correction (6 parameters), spatial smoothing (5-mm full-width-half-maximum Gaussian kernel), high-pass temporal filtering (100 s), and skull stripping. We coregistered participant's functional images to their MBW T2-weighted structural image, which was in turn registered to the MNI-152 average brain. We excluded participants with motion values >2 standard deviations above the group mean as defined by FSL DVARS (54), and verified that each participant had BOLD signal in our regions-of-interest (ROIs). We defined our ROIs as described next (see Figure 1A; ROI centroids in Table S1). Bilateral mPFC and amygdala were defined by 50% probability masks in the FSL Harvard-Oxford atlas. Bilateral rACC was extracted from the Freesurfer atlas. Eigenvariate timeseries from these regions were then extracted to provide us a matrix of size *timepoints* × *ROI* × *subjects* for each of the 3 groups. Please refer to Supplemental Information SI-1.3 for further information.

We next performed hemodynamic deconvolution (55) on ROI timeseries to minimize the confound of intra-subject variability of the hemodynamic response function (HRF). The necessity for this step was that fMRI is an indirect measure of neural activity; and the HRF represents that component of fMRI that is non-neural in origin. Given that HRF varies spatially across the brain, as well as across individuals (56), deconvolution minimizes this confound unique to fMRI. Deconvolution also helps in achieving improved estimation of effective connectivity (57), and minimizing potential confounds resulting from HRF variability within and between individuals (whether normal or pathologic). The deconvolution algorithm, based on the Cubature Kalman filter and Smoother (CKF-S) (58, 59), jointly estimates latent neural timeseries and the ROI-specific HRF. The technique has been extensively used in recent works [for example, see (58, 59)]. The deconvolution step was performed in the Matlab R2013b platform using a publicly available toolbox (http://users.ugent.be/~dmarinaz/HRF_deconvolution.html), with both inputs and outputs being matrices of size *timepoints* × *ROI* × *subjects* for each group.

### Effective connectivity analysis

Dynamic effective connectivity (DEC), a measure that provides the directional connectivity value between pairs of regions at every time instant, was evaluated between all ROI pairs by employing Kalman-filter based time-varying Granger causality (41). Granger causality (GC) is a technique used to study causal functional relationships between brain regions. The underlying concept holds that if past values of a timeseries can predict the future values of another timeseries, a causal influence from the former to the latter can be inferred (41). As in prior studies (58, 59), the deconvolved timeseries from each ROI and each participant (using the *timepoints* × *ROI* × *subjects* matrix) were input to a dynamic multivariate autoregressive (dMVAR) model for estimating effective connectivity between the ROIs, which was solved in a Kalman-filter framework. The dMVAR model coefficients vary as a function of time, giving us the DEC timeseries for every connection. DEC length was identical to the number of time points in the fMRI data; that is, for every connection, one DEC value was obtained for every time point. Using DEC, we obtained the desired block-specific connectivity values (60). Specifically, the time points associated with those trials of the fearful-face stimulus for each of the 3 fearful-face blocks were identified and the DEC values corresponding to those periods were extracted separately for each block, for every connection.

We then sought to identify those connectivities that increased significantly across the blocks, in accordance with our hypotheses, indicating enhanced engagement and contributing to effective top-down modulation and emotion regulation (32). Our hypotheses specifically pertained to the block-to-block within-group increases in connectivity, with higher connectivity corresponding to enhanced engagement (33). Increasing engagement across successive blocks is desirable since increased fronto-limbic connectivity is associated with better top-down modulation and emotion regulation (32). We therefore expected that prefrontal and limbic systems would need to progressively increase in synchronization in order to achieve top-down modulation, which would be reflected in our analyses as progressive increases in effective connectivity. Hence, we specifically looked for connections exhibiting this DEC profile: block3 > block2 *and* block2 > block1 (ANCOVA followed by pairwise *t*-tests, *p* < 0.05, Bonferroni corrected. Analyses were controlled for motion, age, sex, and education). This procedure was performed separately for the three groups to test our hypotheses. We additionally performed exploratory analyses of connectivity between the rACC and the amygdala and mPFC, given its proven involvement during fearful face processing (32). We followed this with between-groups comparisons (ANCOVA followed by *T*-tests, *P* < 0.05 Bonferroni corrected). It is important to note that progressive increases in DEC are independent of changes in brain activation, since effective connectivity modeling normalizes absolute signal strengths in its formulation (41). Hence, connectivity and activation findings are mutually exclusive, and this holds true at the population level as well. Associations between significant between-group connectivity values (as dependent variables) with relevant clinical variables (as independent variables) were tested through linear regression (SFR, Eating Disorder Examination [EDE] and its “*shape concerns*” subscale, BDD Yale-Brown Obsessive-Compulsive Scale (BDD-YBOCS), and Hamilton anxiety rating scale [HAMA]) (*P* < 0.05, Bonferroni corrected for 4 comparisons).

## Results

### Demographics and task behavior

The three groups did not differ significantly on age, sex or years of education (Table 1). Please refer to Supplemental Information SI-2.1 for details about comorbidities.

**Table 1:** Demographics and psychometric scores.

**Characteristic**	**AN group (*****N*** = **25)**		**BDD group (*****N*** = **32)**		**Control group (*****N*** = **37)**		***P*-value**
	**Mean**	**S.D**.	**Mean**	**S.D**.	**Mean**	**S.D**.	
Age (years)	22.1	4.5	23.5	4.8	21.7	4.5	NS
Female/male	23/2		27/5		32/5		NS^†^
Education (years)	14.1	3.1	14.9	3.2	13.9	2.7	NS
BMI	20.3^b^	1.4	22.5^a^	3.2	22.3^c^	3.0	**P** < 0.01
EDE global score	2.8	1.3	N/A	–	N/A	–	–
YBC scores	20.5	7.9	N/A	–	N/A	–	–
BDD–YBOCS score	N/A	–	29.5	5.5	N/A	–	–
HAMA score	7.0^a^	6.0	10.1^a^	6.7	2.2^b^	1.8	**P** < 0.001
MADRS score	10.4^a^	9.3	15.5^a^	7.9	1.3^b^	1.7	**P** < 0.001
BABS score	10.8	6.7	15.2	3.2	N/A	–	**P** < 0.01
Duration of illness (months)	80.9	61.9	120.3	71.9	N/A	–	**P** < 0.05
Lowest lifetime BMI	15.9	1.6	N/A	–	N/A	–	–
BDD appearance concerns (number in each category) AN subtype	Restricting type: 24 Binge eating /purging type: 1		Face only: 9 Non face only: 1 Face /non-face: 22				

BDD, body dysmorphic disorder; BDD-YBOCS, BDD version of the Yale–Brown Obsessive-Compulsive Scale; BABS, Brown Assessment of Beliefs Scale; BMI, body mass index; EDE, Eating Disorder Examination; HARS, Hamilton anxiety rating scale; MADRS, Montgomery-Åsberg Depression Rating Scale; N/A, not applicable; S.D., Standard deviation; YBC, Yale-Brown-Cornell eating disorder scale.

^a, b, c^Letters indicate which groups differed significantly on the ANOVA follow-up tests

*†χ^2^ test was performed to test for female/male*.

With the omnibus ANOVA test on reported subjective fear ratings (SFR), we found a significant effect of task block [*F*_(2, 274)_ = 4.89, *P* = 2.3 × 10^−4^], but no interaction effect between group and block [*F*_(8, 274)_ = 1.37, *P* = 0.21]. Additionally, the groups did not differ significantly on SFR [*F*_(2, 91)_ = 2.63, *P* = 0.08], or the shape of scrambled faces [*F*_(2, 91)_ = 1.20, *P* = 0.31]. Given the trend for group differences, we performed pairwise comparisons with SFR. There were no significant differences between AN and BDD, or BDD and HC, but elevated subjective fear was observed in AN compared to HC (*T* = 2.43, *P* = 0.018).

For the analysis of changes in SFR across the 3 fearful face blocks, we found significant within-group change in all groups [AN: *F*_(2, 72)_ = 0.95, *P* = 3.3 × 10^−4^; BDD: *F*_(2, 93)_ = 1.16, *P* = 1.1 × 10^−4^; HC: *F*_(2, 108)_ = 0.51, *P* = 0.016]. With follow-up *T*-tests we found that, independently within each group, SFR significantly increased from block-1 to block-2 (AN: *T* = 3.45, *P* = 0.001; BDD: *T* = 3.64, *P* = 5.6 × 10^−4^; HC: *T* = 2.59, *P* = 0.011), and decreased from block-2 to block-3 (AN: *T* = −3.40, *P* = 0.001; BDD: *T* = −3.34, *P* = 0.001; HC: *T* = −2.15, *P* = 0.035 [not significant with Bonferroni correction]).

### Effective connectivity results

Figure 1B shows the significant connections identified as increasing progressively across the three fearful-face blocks within each group (see *F*-value, *T*-value, and *p*-value tables in Supplemental Information SI-2.2). We found evidence supporting our hypotheses. First, significant mPFC-amygdala bidirectional connectivity was identified only in HCs. Second, a significant unidirectional mPFC-to-amygdala connectivity was identified only in BDD; and third, no connectivity reached the level of statistical significance between prefrontal regions and amygdala in AN. Additionally, significant rACC-to-amygdala connectivity was identified only in BDD, and significant rACC-to-mPFC connectivity was identified only in AN.

**Figure 2:**
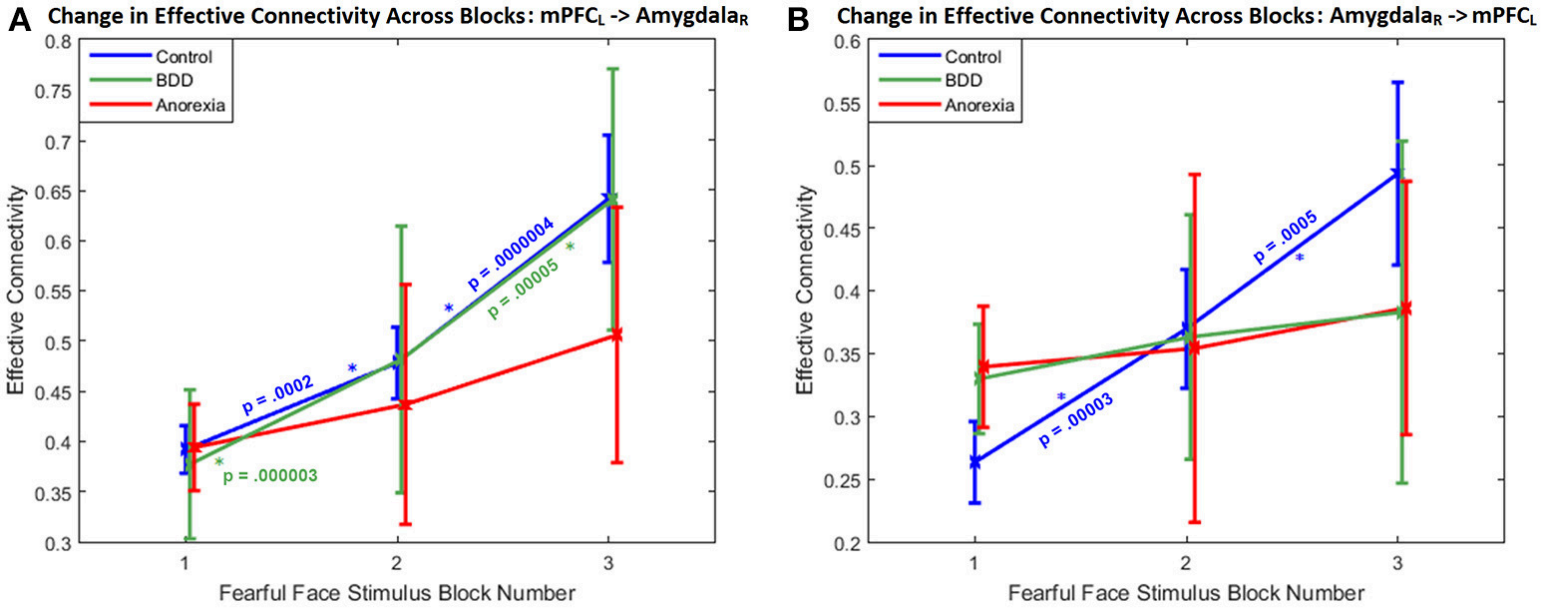
Change in effective connectivity across blocks: The bidirectional connectivity profile that distinguished the three groups: within-group connectivity between the left mPFC and the right amygdala shown across the three successive task blocks, whose enhanced engagement for fronto-limbic modulation was found to differentiate between AN, BDD, and controls. **(A)** With the mPFC-to-amygdala connectivity, a monotonically increasing trend is apparent, with the change being significantly large (*p* < 0.05, Bonferroni corrected) in HCs and in BDD, but not in AN. It is also noticeable that the variability monotonically increases from block1 through block3 in all the groups, suggesting increased inter-subject variability in fronto-limbic engagement as the blocks progress. **(B)** With the amygdala-to-mPFC connectivity, a monotonically increasing trend is apparent only in HCs, with the change being significantly large (*p* < 0.05, Bonferroni corrected). *p*-values in the figure correspond to within-group block-to-block comparisons.

Regarding the within-group changes in connectivity across successive task blocks, the bidirectional connectivity profile between the left mPFC and the right amygdala was distinct among the three groups; specifically, the mPFC-to-amygdala connectivity differentiated participants with AN from BDD and HCs (which was significant in HC and BDD but not AN) (Figure 2A), whereas the amygdala-to-mPFC connectivity differentiated BDD and AN from HCs (which was significant in HC but not in the clinical groups) (Figure 2B).

Between groups, there was a significant difference in mPFC–to-amygdala connectivity [3-way ANCOVA: *F*_(3, 91)_ = 4.0459, *P* = 0.0095]. AN showed weaker mPFC–to-amygdala connectivity compared to HCs (*T* = 3.18, *P* = 0.0015, Cohen's *d* = 0.18) and BDD (*T* = 2.82, *P* = 0.0050, Cohen's *d* = 0.17) (Figure 2A). In BDD, while the top-down fronto-limbic effective connectivity network, as compared to AN, more resembled the network in HCs (Figure 1B), we found abnormalities suggesting that BDD participants might employ alternate strategies to facilitate a response to repeated fearful faces. For comparison, we also analyzed between-group differences in connectivity during viewing of scrambled faces, and found no significant between-group aberrations [3-way ANCOVA, *F*_(3, 91)_ = 0.47, *P* = 0.24]. Please refer to Supplemental Information SI-2.2 for a detailed presentation and discussion of the changes in effective connectivity across blocks for the remaining significant connections.

### Exploratory activation analyses

Although the goal of this study was to investigate changes in fronto-limbic directional *connectivity* with repeated exposure to fearful faces, which is independent of the degree of *activation* represented in the BOLD signal, as a supplementary analysis we extracted eigenvalues of time series activations in the ROIs to examine patterns of change across the three blocks. (See Supplemental Information SI-1.4 for additional details of the ROI eigenvariate activation analyses.) HC exhibited a trend of decreasing right amygdala activation across the three fearful face blocks (*P* = 0.097, *T* = −1.7, Cohen's *d* = 0.22), which was not observed in the AN (*P* = 0.65) or BDD (*P* = 0.23) groups, although there were no significant differences between groups. There were no significant increases or decreases in the mPFC or rACC in any group.

### Associations with clinical measures

**Figure 3:**
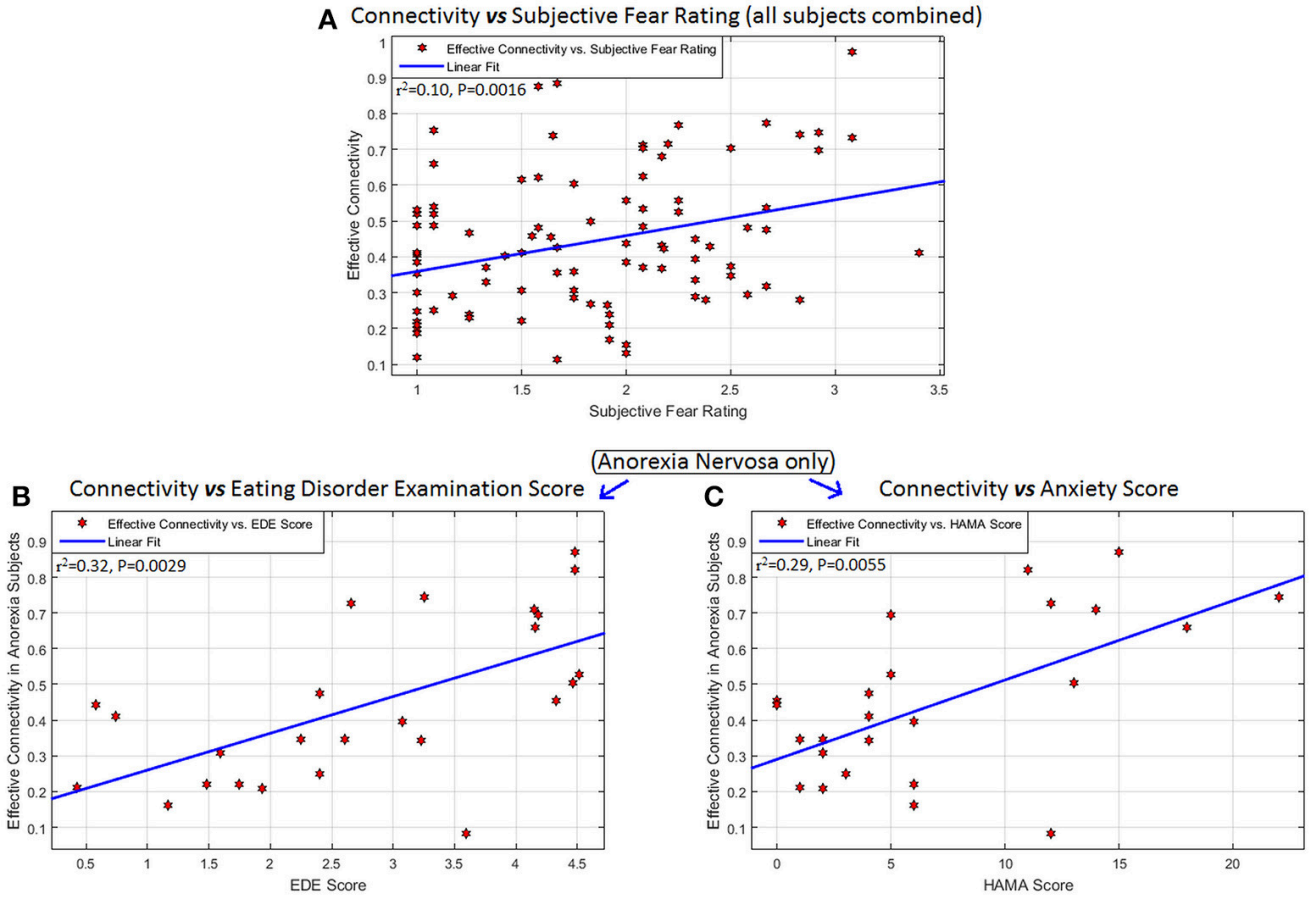
Associations between connectivity and behavioral measures: Behavioral/clinical relevance of the connectivity from the left mPFC to the right amygdala: significant association of this connectivity (averaged across all task blocks) with **(A)** subjective fear rating across all participants (*R*^2^ = 0.10, *P* = 0.0016, 95% CI = [0.02,0.24]), **(B)** Eating Disorder Examination score (EDE) in anorexia nervosa (*R*^2^ = 0.32, *P* = 0.0029, 95% CI = [0.05,0.62]), and **(C)** Hamilton Anxiety Scale (HAMA) in anorexia nervosa (*R*^2^ = 0.29, *P* = 0.0055, 95% CI = [0.03,0.59]). No other significant associations were found between any significant connection and any of these scores, as well as with scales measuring obsessive and compulsive eating disorder and BDD symptoms (Yale-Brown-Cornell Eating disorder scale and the body dysmorphic disorder version of the Yale-Brown Obsessive-Compulsive scale, respectively) and the Montgomery-Asberg Depression Rating Scale (MADRS).

The left mPFC -to- right amygdala connectivity (averaged across all fearful-face task blocks) had significant association with SFR across all participants (*R*^2^ = 0.10, *P* = 0.0016, 95%-CI = [0.02,0.24]), with EDE in AN (*R*^2^ = 0.32, *P* = 0.0029, 95%-CI = [0.05,0.62]), and with HAMA in AN (*R*^2^ = 0.29, *P* = 0.0055, 95%-CI = [0.03,0.59]) (Figure 3). Given that EDE and HAMA scores were themselves correlated in AN (*r* = 0.53, *P* = 0.0061), we performed a partial correlation analysis between connectivity and these measures and found that mPFC-to-amygdala connectivity in AN had a significant association with the EDE score after controlling for HAMA (*r* = 0.41, *P* = 0.0485), and with HAMA controlling for EDE (*r* = 0.42, *P* = 0.0440). Thus, eating disorder symptom intensity, and severity of anxious symptoms as well, showed independent associations with mPFC-to-amygdala connectivity in AN, wherein higher symptom severity was associated with stronger connectivity.

There were no significant associations between any other combinations of connectivity and clinical measures, including EDE “shape concerns” subscale, BDD-YBOCS, lowest lifetime BMI, current BMI, and duration of illness.

## Discussion

This is the first study to investigate and compare the connectivity patterns of emotion processing in AN and BDD, conditions that share aberrant body image as a core diagnostic feature. There are four major findings:

1. As hypothesized, we found significantly enhanced engagement of within-group bidirectional mPFC-amygdala connectivity only in HCs during repeated exposure to fearful faces; indicating that enhanced top-down engagement as well as bottom-up feedforward signaling are characteristic of the response to repeated fearful face stimuli in healthy individuals.
2. In accord with our hypothesis, while we found enhanced engagement of within-group mPFC-to-amygdala connectivity in BDD, the amygdala–to-mPFC connectivity did not exhibit significantly enhanced engagement; indicating successful top-down engagement but impaired bottom-up feed-forward signaling, consistent with other evidence of limbic hypo-responsiveness in this disorder (20).
3. Also in line with our hypothesis, both mPFC-to-amygdala and amygdala-to-mPFC within-group connectivity upon repeated exposure to fearful faces were nonsignificant in AN, indicating insufficient communication between these regions. Additionally, AN participants showed significantly weaker mPFC-to-amygdala connectivity (between-group comparison) compared to HC and BDD, indicating a lower degree of top-down modulation in AN. The specificity of this to fearful face processing is supported by the absence of significant group differences in connectivity for the scrambled faces control task.
4. There were significant associations between the mPFC-to-amygdala connectivity and subjective fear rating in all participants, and with eating disorder and anxiety symptom intensity in AN participants, underscoring the behavioral and clinical relevance of this connection.

In summary, our experimental design of having participants reflect upon, and subjectively rate, their level of fear during fearful face viewing was intended to instantiate the top-down modulation of emotion (37, 38). In this context, the results indicate evidence of both shared and unique abnormal fronto-limbic fear circuitry in these two clinical phenotypes.

In both the clinical groups, we found, as hypothesized, abnormal connectivity between mPFC and amygdala. These regions are integral to the expression and modulation of fear, which are facilitated through their connectivity (35). It is well known that mPFC plays a central role in emotion regulation, and is likely to engage with the amygdala during repeated exposure of a negatively valenced stimulus (32). Consistent with this, HCs displayed progressively increasing bidirectional mPFC-amygdala connectivity, indicating rising efficiency of two-way communication (akin to increasing harmonization). This was not the case, however, in both clinical groups.

As depicted in Figure 2, communication from the mPFC to the amygdala, and from amygdala to mPFC, is markedly deficient in AN. This is consistent with, and bridges, two bodies of literature: the prominence of anxious traits well in advance of the onset of weight loss and other clinical indicators in AN (15), and evidence of connectivity defects in anxiety disorders (31). Weak mPFC-to-amygdala connectivity (33), has been observed in those with high trait anxiety (61). Interestingly, reduced mPFC-amygdala connectivity has also been observed in autism (62), whose risk architecture may partially overlap with AN (2).

We also observed evidence in both AN and BDD that signals originating in the amygdala may not be instantiating feed-forward information to the mPFC, as evidenced by the lack of significant amygdala-to-prefrontal connectivity. As regards BDD, this may account for previously-observed limbic hypo-responsiveness in participants with BDD during exposure to their own faces, despite their rating of the experience as aversive (20). While significant mPFC-to-right-amygdala connectivity during repeated fearful faces suggests an intact top-down modulation in the BDD group, the amygdala-to-prefrontal connections were weaker, suggesting blunted feed-forward signaling. The observation that CBT treatment utilizing exposure techniques, requiring the engagement of top-down connections like mPFC-to-amygdala (63), is at least moderately beneficial in BDD (42, 43), raises the possibility that the important direction in this two-way connection for success of the treatment may primarily be top-down modulation.

We next discuss the connections that were significantly engaged in AN and/or BDD but not in HC. The rACC-to-mPFC connection, which exhibited increased engagement in AN but not in HC or BDD, is known to be elevated during situations of conflict (64). Hence, its elevated engagement might relate to the AN participants' efforts to compensate for their inability in responding adequately and conventionally to repeated fearful faces. We also observed enhanced engagement of rACC-to-amygdala connectivity in BDD. This connection often emerges in response to impaired prefrontal-amygdala connectivity: Kujawa et al. (65) found this connectivity to be associated with impaired fronto-limbic connectivity during viewing of emotional faces in those with anxiety disorders. We suggest that the increased engagement of this connection is an attempt by the brain to engage alternate strategies to compensate for impaired amygdala-to-mPFC connectivity.

Although we hypothesized that mPFC-to-amygdala connectivity would be associated with intensity of anxiety in both clinical groups, it appears only in AN. Increases in this connectivity were associated with an increase in anxiety and with total EDE score. What the association of this connectivity with EDE total score (Figure 3B) and intensity of anxiety (Figure 3C) implies clinically remains uncertain. A parsimonious explanation is that the failure of top-down modulation influences the expression of causal processes that culminate in symptom morbidity in eating disorder and anxiety domains, and that the two are interdependent, or synergistic. One possible explanation of this finding is that those AN participants who had higher anxiety and/or more severe eating disorder symptoms could have required greater engagement of the mPFC-to-amygdala connectivity to respond adequately to repeated fearful face stimuli; yet, even considering those with greater engagement, generally ineffective connectivity in this group still results in average connectivity strength being lower than in controls. No studies have assessed the association between prefrontal-amygdala connectivity and anxiety as measured by HAMA scores, so this suggestion remains conjectural.

Further, the association of the mPFC-to-amygdala connectivity with SFR across all the participants (Figure 3A) implies that this connection is a consequence of (or, alternatively, contributes to) the subjective experience of fear, independent of any psychopathological syndrome. Greater connectivity corresponded to higher subjective fear, implying enhanced engagement with elevated fear response. Anxiety and fear are different constructs, mediated by different neural pathways (32). The mPFC-to-amygdala connectivity was associated with both fear and anxiety only in AN, hence impaired processing of fear may be associated with anxiety in AN but not in BDD.

While bilateral ROIs were considered in this work, the connectivity findings were not entirely symmetric. In controls, the left mPFC was involved in bidirectional connectivity with the amygdalae. In the BDD group, the *left mPFC -to- right amygdala* connection was identified. The right mPFC was not involved in the fronto-limbic network in any of the groups (Figure 1). From these observations, one could infer that the left mPFC is necessary for fearful face processing. Our findings corroborate prior research, which has found robust evidence for the involvement of the left mPFC in fear processing and emotion regulation (32).

We observed lateralization in amygdala connectivity as well. While bilateral amygdalae exhibited bidirectional connectivity with the left mPFC in controls (Figure 1), only the right amygdala was connected with the mPFC in BDD (*left mPFC -to- right amygdala*). We did predict this prefrontal-to-amygdala connectivity in BDD, because cognitive behavioral therapy (CBT) techniques, which utilize exposures, are at least moderately beneficial in BDD (42, 43). The fact that the right amygdala alone was involved in the BDD network, coupled with the moderately beneficial response of BDD individuals to CBT treatment, hints that the involvement of right amygdala alone might be sufficient to avert dysfunctional top-down response to repeated fearful faces. In agreement with this, prior studies have found the right amygdala to mediate fear processing to a greater degree than the left amygdala (34).

The exploratory activation analysis revealed a trend for reduced right amygdala activity across blocks for the HC but not AN or BDD groups, although differences between groups were non-significant. The goal of this study was to investigate changes in fronto-limbic directional connectivity with repeated exposure to fearful faces in AN, BDD, and healthy controls. Connectivity, as a concept, measures the interrelationship between fMRI activities of two distinct brain regions. With both functional and effective connectivity, the connectivity value is independent of the degree of activation as observed from the fMRI signal, and is sensitive only to the variations observed in the two time series. Specifically, effective connectivity modeling normalizes absolute signal strengths (41), and hence progressive increases in connectivity are independent of changes in brain activation. This holds true at population level as well. While the finding of a trend for decreasing right amygdala activation in HC is not significant, it nonetheless suggests a pattern of reducing amygdala activation with repeated fearful faces. An important point to note is that this experiment was not designed to test habituation. Rather, we designed the task to test fronto-limbic communication engaged by internal labeling of one's emotional state. The task, as opposed to passive-viewing or gender labeling, would therefore be expected engage modulation of limbic regions. This would therefore likely dilute face-to-face or block-to-block trends in activation (66, 67). Moreover, this experiment used all unique face stimuli rather than repeated presentation of the same face, which likely further diminished habituation effects.

In a previous study in AN examining activation to fearful faces, Cowdrey et al. (40) found that there were no significant differences between AN and HC while viewing fearful faces and labeling their gender. In the current study, we also did not find any significant activation differences during viewing of fearful faces, but rather significant connectivity differences.

The activation findings in the current study thus may have important heuristic value in the context of the primary connectivity results: enhanced bidirectional mPFC-amygdala connectivity in controls with successive blocks of fearful faces, only significant mPFC-to-amygdala connectivity in BDD, and no significant fronto-limbic connectivity in AN. Since fronto-limbic effective connectivity patterns were associated with both immediate subjective reports of fearfulness across all participants as well as longer-term symptom profiles related to anxiety and eating disorder severity (in the AN group), this provides empirical support for the idea that connectivity and its dynamics in these circuits may be more clinically relevant and a more direct measurement of important neural communication than the simple regional activation patterns.

Concerning subjective fear findings, although there were no significant group differences averaged across trials (only at trend level, driven by higher subjective fear in AN compared to HC), of more interest was the observation that all groups exhibited increased subjective fear from block-1 to block-2 and decreased SFR from block-2 to block-3. This suggests a pattern of increased subjective fear during the first part of the experimental run when they were introduced to emotionally arousing stimuli, and decreased subjective fear during the second part when fronto-limbic regulation might have had an increasing impact. If fronto-limbic connectivity and subjective fear are linked, this possibly indicates a time lag between the initial increase in connectivity (since it significantly increases from block 1 to block 2, at least in controls and BDD) and subsequent subjective experience of fear. Why the AN group also exhibited this pattern despite not showing elevated fronto-limbic engagement during the same task blocks is unclear. This mismatch between connectivity and subjective experience might be a cause or consequence of alexithymia (68, 69); wherein neural correlates do not match with self-reported experiences related to emotion in AN. This aspect demands further attention in future studies. Please refer to Supplemental Information SI-2.4 for a discussion on associations between connectivity and behavior.

This work contributes to a mechanistic understanding of the neural substrates underlying the similarities and differences in AN and BDD, and may have important clinical relevance regarding treatments that engage fronto-limbic circuitry. As regards the clinical therapeutic implications of these data, weak connectivity to fearful stimuli in both clinical groups may explain, at least partially, why treatments that use exposure and response prevention (ERP), a type of CBT, have relatively small effects in AN (44), and have modest benefits in BDD (43). Taken together, this raises the possibility that ERP, which has theoretical links to animal research in fear extinction (70), on average may show low effectiveness for treatment of AN; to a lesser degree the same may be true for BDD, since the underlying aberrant fronto-limbic connectivity may limit its potential effectiveness. However, the considerable variance observed in the current study has implications for identifying individuals who might respond better to an exposure based therapies. Admittedly speculative, a possibility for enhancing treatment could be a pharmacotherapeutic agent that could selectively increase activity within the serotonergic inhibitory neurons that project from the mPFC to the amygdala and reduce activation within the “aversive amplification” circuit (71). Please refer to Supplemental Information SI-2.3 for further discussion points.

Finally, we discus several limitations and future directions. (i) Our AN cohort consisted of weight-restored individuals, in the interest of minimizing the potential confounding effect of weight. Consequently, our findings cannot necessarily be extrapolated to underweight individuals. (ii) We did not acquire participants' emotional ratings of the faces (rather, self-reported fear), which may be relevant given the possibility of misinterpretation of emotional expressions that have been observed in BDD (72) and weight-restored adolescents with AN (73). We acknowledge that offline testing of affect recognition would have been beneficial. (iii) Comorbid AN and BDD (CAB) is not uncommon; however, we excluded those with this comorbidity pattern. Studying brain networks in CAB could shed light on the common and distinguishing neural signatures of CAB in relation to the individual disorders. The mechanistic models and treatment regimens of the disorder that is closer to CAB could then be considered more relevant to CAB psychopathology. (iv) We allowed comorbid diagnoses to ensure that we included a representative clinical sample, although this may have confounded our findings. (v) Our analyses were hypothesis-driven; while exploratory analyses of mPFC and amygdala seed-to-whole brain connectivity might have yielded insights about additional brain communication patterns. However, performing deconvolution and DEC on such a large scale is computationally unwieldy at present even using computing clusters. (vi) The sample sizes of the three groups were different, and within-group statistics cannot control for this. We have provided effect sizes to help the reader develop a more complete picture, which suggests that sample sizes had minimal impact on our findings. (vii) Future research would need to determine how these findings of abnormal connectivity might contribute to the development of AN and BDD, or if they are secondary effects of the illness itself or other pathophysiological processes. (vii) Another important future direction includes directly studying the relationship between abnormal connectivity and treatment response.

## Author contributions

DR, CB, MS, and JF: literature search; DR, CB, SK, MS, and JF: study design; KL, TM, SK, MS, and JF: data collection; DR, CB, TM, FM, and JF: data analysis; DR, MS, and JF: data interpretation; DR: figures; DR, CB, SK, MS, and JF: writing.

### Conflict of interest statement

The authors declare that the research was conducted in the absence of any commercial or financial relationships that could be construed as a potential conflict of interest.
