# Supplementary material for: Aberrant Dynamic Connectivity for Fear Processing in Anorexia Nervosa and Body Dysmorphic Disorder
Source: Front Psychiatry. 2018 Jun 26;9:273. doi: 10.3389/fpsyt.2018.00273 (PMC6028703; doi:10.3389/fpsyt.2018.00273)
Supplement: Supplementary file 1 [file Data_Sheet_1.PDF]

SUPPLEMENTAL INFORMATION

Aberrant dynamic connectivity for fear processing in anorexia nervosa and body dysmorphic disorder

D Rangaprakash[1], Cara Bohon[2], Katherine E. Lawrence[1], Teena Moody[1], Francesca Morfini[1], Sahib S. Khalsa[1,3,4], Michael Strober[1], Jamie D. Feusner[1]

[1] Department of Psychiatry and Biobehavioral Sciences, University of California Los Angeles, Los Angeles, CA, USA

[2] Department of Psychiatry and Behavioral Sciences, Stanford University, Stanford, CA, USA

[3] Oxley College of Health Sciences, University of Tulsa, Tulsa, OK, USA

[4] Laureate Institute for Brain Research, University of Tulsa, Tulsa, OK, USA

SI-1. Supplemental Methods

SI-1.1. Participants

Recruitment: Participants were recruited through the University of California Los Angeles and surrounding communities using the following: (i) referrals from mental health providers, dermatologists, and cosmetic surgeons, (ii) via posted advertisements and flyers, and (iii) a website introducing the research. Forty BDD, 30 weight-restored AN, and 41 healthy controls (HC) were originally recruited in total. Some of the participants were excluded, or their data was excluded, due to the reasons mentioned as follows: (i) excluded 8 BDD (3 errors in data acquisition, 2 excessive motion, and 3 being under medication); (ii) excluded 5 AN (3 not weight-restored, 1 errors in data acquisition, and 1 being under medication); and (iii) excluded 4 HC (3 errors in data acquisition and 1 excessive motion). This left us with 32 BDD, 25 AN, and 37 HC participants, from whom usable data was finally obtained.

SI-1.2. Fearful Face Task

Face stimuli were acquired from the Macbrain database, the University of Pennsylvania Facial Emotional, and the Psychological Image Collection at Stirling (http://pics.psych.stir.ac.uk/). We used MacStim 3.0 (White Ant Occasional Publishing) to program stimuli presentation and to record subjective fear ratings. The shapes of the scrambled faces (round or oval) were randomized across trials.

SI-1.3. Functional Data Preprocessing

Upon fMRI data preprocessing, we extracted eigenvariate time series from each of the regions of interest (ROIs). This choice was motivated by the fact that other measures such as percentage signal change are based on homogenous averaging across voxels (Friston, Rotshtein, Geng, Sterzer, & Henson, 2006), which could "dilute" the signal of interest when an ROI contains both activated and deactivated voxels. Fig.1a in main text showed the 6 ROIs on a 3D brain; here we provide the centroids of all 6 ROIs (Table S1).

Table S1. Centroid locations of our regions-of-interest in the MNI space: bilateral medial prefrontal cortex (mPFC), rostral anterior cingulate cortex (rACC) and amygdala.

	**MNI coordinates**		
	**x**	**y**	**z**
**mPFC L**	-4	44	-18
**mPFC R**	4	44	-18
**rACC L**	-6	36	2
**rACC R**	6	38	4
**Amygdala L**	-22	-4	-18
**Amygdala R**	24	-4	-18

SI-1.4. ROI activation analysis

In order to draw broader inferences about the degree that each region is activated (as well as connected) with repeated exposure to fearful faces, as a supplementary investigation we performed an fMRI activation analysis in bilateral mPFC, rACC and amygdala using FSL software package's FEAT tool (https://fsl.fmrib.ox.ac.uk/fsl/fslwiki/FEAT).

Specifically, we performed GLM analysis with the three first-level regressors of interest being the three fearful face stimuli blocks. Six motion parameters were included as regressors of

no interest. In the second-level, we probed the contrast that quantified increasing activation across the three blocks, and the contrast that quantified decreasing activation across the three blocks. Statistics were performed through permutation testing, using the “randomize” utility in FSL (https://fsl.fmrib.ox.ac.uk/fsl/fslwiki/Randomise). Eigenvalues were extracted for each of the six ROIs for each of the groups. Within-group comparisons were performed for each group separately (comparing eigenvalues against 0). Between-group comparisons were performed using the eigenvalues of each group.

SI-2. Supplemental Results and Discussion

SI-2.1. Participant Characteristics

Comorbidities: In the AN group, 8 participants had comorbidities: 4 had comorbid generalized anxiety disorder (GAD), 2 had major depressive disorder (MDD), and 2 had GAD and MDD. Twenty-four AN participants met DSM-IV criteria for restricting type. One had binge eating/purging type, and was included in the final analysis after confirming that this person’s connectivity findings were not an outlier. Among BDD participants, 17 had comorbidities: 7 had MDD, 2 had dysthymic disorder, 1 had GAD, 1 had agoraphobia, 1 had social phobia, 1 had panic disorder with agoraphobia, 1 had MDD and GAD, 1 had MDD and social phobia, 1 had social phobia and GAD, and 1 had MDD, GAD, and social phobia. In the BDD group, 9 had facial concerns, 1 had non-facial concerns, and 22 had facial as well as non-facial concerns.

Consistent and reliable information about the duration of time that AN participants were weight-restored was not available as this information was not known to the majority of

individuals. This is likely due to the fact that the majority were recruited from the community rather than long-term inpatient or residential treatment facilities, and treatment providers (therapists, psychiatrists, and dieticians) frequently do not inform the patients of their weight during recovery and encourage them not to check their own weight. However, lowest BMI and illness duration data were available.

SI-2.2. Change in Effective Connectivity Across Task Blocks: T-statistic, P-value and Effect Size Tables

This section provides the statistics (F-value, T-values, p-values and effect sizes) for the results presented in the main text. In each case, an ANOVA was first performed (F-value in tables), which was followed by independent pairwise t-tests (T-value and p-value in tables). Specifically, we present the following:

1. Statistics for the within-group change in effective connectivity across the three successive fearful-face task blocks:
   - In the control group (**Table S2**).
   - In the BDD group (**Table S3**).
   - In the AN group (**Table S4**).

2. Statistics for the between-group differences in effective connectivity:
   - Between the control and the BDD groups (**Table S5**).
   - Between the control and the AN groups (**Table S6**).

Table S2. *Statistics for the* ***within-group*** *change in effective connectivity across the three successive fearful-face task blocks, in the* ***control*** *group:* ***(a)*** *F-values for block3 > block2 > block1 comparison,* ***(b)*** *T-values for block3 > block2 comparison,* ***(c)*** *T-values for block2 > block1 comparison,* ***(d)*** *p-values for block3 > block2 comparison,* ***(e)*** *p-values for block2 > block1 comparison,* ***(f)*** *effect sizes (Cohen's d) for block3 > block2 comparison,* ***(g)*** *effect sizes for block2 > block1 comparison. Those marked in red were statistically significant (p<0.05, Bonferroni corrected), while those in gray were not. The direction of the connections were from the regions mentioned in the rows to those in the columns, as indicated by the red arrow. T-value threshold corresponding to p<0.05 (Bonferroni corrected) is 3.16. Abbreviations: rACC = rostral anterior cingulate cortex; mPFC = medial prefrontal cortex; amyg = amygdala.*

F-value- Controls: block3 > block2 > block1

→	Amyg_L	Amyg_R	rACC_L	rACC_R	mPFC_L	mPFC_R
**Amyg_L**	0	0.27	0.50	0.52	**5.30**	0.69
**Amyg_R**	0.17	0	0.15	0.13	**3.88**	0.83
**rACC_L**	0.16	0.54	0	0.32	0.84	1.21
**rACC_R**	0.08	0.35	0.81	0	0.08	0.76
**mPFC_L**	**6.20**	**5.26**	**5.76**	0.47	0	1.12
**mPFC_R**	0.00	0.08	0.04	0.01	0.04	0

(a)

T-value- Controls: block3 > block2

→	Amyg_L	Amyg_R	rACC_L	rACC_R	mPFC_L	mPFC_R
**Amyg_L**	0	1.18	0.92	1.67	**4.96**	1.81
**Amyg_R**	0.86	0	0.81	0.68	**3.52**	1.78
**rACC_L**	0.70	2.29	0	1.00	0.99	2.79
**rACC_R**	0.51	0.85	0.55	0	0.32	1.72
**mPFC_L**	**5.34**	**5.12**	**4.99**	1.54	0	2.68
**mPFC_R**	0.06	0.43	-0.02	0.11	0.46	0

(b)

T-value- Controls: block2 > block1

	Amyg_L	Amyg_R	rACC_L	rACC_R	mPFC_L	mPFC_R
Amyg_L	0	0.51	1.59	0.62	**3.95**	0.88
Amyg_R	0.51	0	0.52	0.54	**4.25**	1.18
rACC_L	0.72	0.10	0	1.04	2.26	0.81
rACC_R	0.43	1.22	2.36	0	0.67	1.32
mPFC_L	**4.61**	**3.79**	**4.07**	0.73	0	0.96
mPFC_R	0.15	0.69	0.79	0.32	0.33	0

(c)

P-value- Controls: block3 > block2

	Amyg_L	Amyg_R	rACC_L	rACC_R	mPFC_L	mPFC_R
Amyg_L	0	0.24	0.36	0.10	**9.5×10^{-07}**	0.07
Amyg_R	0.39	0	0.42	0.50	**4.7×10^{-04}**	0.08
rACC_L	0.48	0.02	0	0.32	0.32	0.01
rACC_R	0.61	0.40	0.58	0	0.75	0.09
mPFC_L	**1.4×10^{-07}**	**4.4×10^{-07}**	**8.2×10^{-07}**	0.12	0	0.01
mPFC_R	0.95	0.67	0.98	0.91	0.65	0

(d)

P-value- Controls: block2 > block1

	Amyg_L	Amyg_R	rACC_L	rACC_R	mPFC_L	mPFC_R
Amyg_L	0	0.61	0.11	0.53	**9.1×10^{-05}**	0.38
Amyg_R	0.61	0	0.60	0.59	**2.5×10^{-05}**	0.24
rACC_L	0.47	0.92	0	0.30	0.02	0.42
rACC_R	0.67	0.22	0.02	0	0.50	0.19
mPFC_L	**5.2×10^{-06}**	**1.7×10^{-04}**	**5.3×10^{-05}**	0.47	0	0.34
mPFC_R	0.88	0.49	0.43	0.75	0.74	0

(e)

Effect size (Cohen's *d*): block3 vs. block2

→	Amyg_L	Amyg_R	rACC_L	rACC_R	mPFC_L	mPFC_R
**Amyg_L**	0	0.10	0.08	0.15	**0.44**	0.16
**Amyg_R**	0.08	0	0.07	0.06	**0.31**	0.16
**rACC_L**	0.06	0.20	0	0.09	0.09	0.24
**rACC_R**	0.04	0.07	0.05	0	0.03	0.15
**mPFC_L**	**0.47**	**0.45**	**0.44**	0.14	0	0.24
**mPFC_R**	0.01	0.04	0.00	0.01	0.04	0

(f)

Effect size (Cohen's *d*): block2 vs. block1

→	Amyg_L	Amyg_R	rACC_L	rACC_R	mPFC_L	mPFC_R
**Amyg_L**	0	0.04	0.14	0.05	**0.35**	0.08
**Amyg_R**	0.04	0	0.05	0.05	**0.37**	0.10
**rACC_L**	0.06	0.01	0	0.09	0.20	0.07
**rACC_R**	0.04	0.11	0.21	0	0.06	0.12
**mPFC_L**	**0.40**	**0.33**	**0.36**	0.06	0	0.08
**mPFC_R**	0.01	0.06	0.07	0.03	0.03	0

(g)

Table S3. *Statistics for the* ***within-group*** *change in effective connectivity across the three successive fearful-face task blocks, in the* ***body dysmorphic disorder (BDD)*** *group:* ***(a)*** *F-values for block3 > block2 > block1 comparison,* ***(b)*** *T-values for block3 > block2 comparison,* ***(c)*** *T-values for block2 > block1 comparison,* ***(d)*** *p-values for block3 > block2 comparison,* ***(e)*** *p-values for block2 > block1 comparison,* ***(f)*** *effect sizes (Cohen's d) for block3 > block2 comparison,* ***(g)*** *effect sizes for block2 > block1 comparison. Those marked in red were statistically significant (p<0.05, Bonferroni corrected), while those in gray were not. The direction of the connections were from the regions mentioned in the rows to those in the columns, as indicated by the red arrow. T-value threshold corresponding to p<0.05 (Bonferroni corrected)*

is 3.16. Abbreviations: rACC = rostral anterior cingulate cortex; mPFC = medial prefrontal cortex; amyg = amygdala.

F-value- BDD: block3 > block2 > block1

	Amyg_L	Amyg_R	rACC_L	rACC_R	mPFC_L	mPFC_R
Amyg_L	0	0.09	0.84	0.19	0.15	0.91
Amyg_R	0.47	0	0.43	0.75	0.49	0.42
rACC_L	**5.00**	**3.04**	0	0.11	0.04	0.10
rACC_R	0.30	0.48	0.16	0	0.37	0.27
mPFC_L	0.28	**4.57**	**4.94**	0.41	0	1.24
mPFC_R	0.04	0.03	0.03	0.19	0.04	0

(a)

T-value BDD: block3 > block2

	Amyg_L	Amyg_R	rACC_L	rACC_R	mPFC_L	mPFC_R
Amyg_L	0	0.78	2.12	0.88	1.04	1.95
Amyg_R	0.77	0	1.57	2.00	0.87	1.19
rACC_L	**4.65**	**3.71**	0	0.94	0.64	0.30
rACC_R	1.11	2.15	0.74	0	0.93	1.13
mPFC_L	1.29	**4.22**	**4.48**	1.36	0	1.37
mPFC_R	0.18	0.20	-0.10	0.48	0.01	0

(b)

T-value BDD: block2 > block1

	Amyg_L	Amyg_R	rACC_L	rACC_R	mPFC_L	mPFC_R
Amyg_L	0	0.30	0.76	0.74	0.33	1.23
Amyg_R	1.68	0	0.89	1.14	1.74	1.14
rACC_L	**4.38**	**3.43**	0	-1.16	0.01	0.87
rACC_R	0.89	-0.11	0.55	0	1.08	0.52
mPFC_L	0.60	**4.71**	**4.09**	0.77	0	2.59
mPFC_R	0.63	0.47	0.71	1.35	0.74	0

(c)

P-value- BDD: block3 > block2

	Amyg_L	Amyg_R	rACC_L	rACC_R	mPFC_L	mPFC_R
**Amyg_L**	0	0.44	0.03	0.38	0.30	0.05
**Amyg_R**	0.44	0	0.12	0.05	0.38	0.24
**rACC_L**	**4.3×10^{-06}**	**2.4×10^{-04}**	0	0.35	0.52	0.76
**rACC_R**	0.27	0.03	0.46	0	0.35	0.26
**mPFC_L**	0.20	**2.9×10^{-05}**	**9.5×10^{-06}**	0.17	0	0.17
**mPFC_R**	0.86	0.84	0.92	0.63	0.99	0

(d)

P-value- BDD: block2 > block1

	Amyg_L	Amyg_R	rACC_L	rACC_R	mPFC_L	mPFC_R
**Amyg_L**	0	0.76	0.45	0.46	0.74	0.22
**Amyg_R**	0.09	0	0.37	0.26	0.08	0.26
**rACC_L**	**1.5×10^{-05}**	**6.5×10^{-04}**	0	0.25	0.99	0.39
**rACC_R**	0.37	0.91	0.58	0	0.28	0.60
**mPFC_L**	0.55	**3.4×10^{-06}**	**5.2×10^{-05}**	0.44	0	0.01
**mPFC_R**	0.53	0.64	0.48	0.18	0.46	0

(e)

Effect size (Cohen's *d*): block3 vs. block2

	Amyg_L	Amyg_R	rACC_L	rACC_R	mPFC_L	mPFC_R
**Amyg_L**	0	0.07	0.20	0.08	0.10	0.18
**Amyg_R**	0.07	0	0.15	0.19	0.08	0.11
**rACC_L**	**0.44**	**0.35**	0	0.09	0.06	0.03
**rACC_R**	0.11	0.20	0.07	0	0.09	0.11
**mPFC_L**	0.12	**0.40**	**0.42**	0.13	0	0.13
**mPFC_R**	0.02	0.02	-0.01	0.05	0.00	0

(f)

Effect size (Cohen's *d*): block2 vs. block1

→	Amyg_L	Amyg_R	rACC_L	rACC_R	mPFC_L	mPFC_R
**Amyg_L**	0	0.03	0.07	0.07	0.03	0.12
**Amyg_R**	0.16	0	0.08	0.11	0.16	0.11
**rACC_L**	**0.41**	**0.32**	0	-0.11	0.00	0.08
**rACC_R**	0.08	-0.01	0.05	0	0.10	0.05
**mPFC_L**	0.06	**0.44**	**0.39**	0.07	0	0.25
**mPFC_R**	0.06	0.04	0.07	0.13	0.07	0

(g)

Table S4. *Statistics for the* ***within-group*** *change in effective connectivity across the three successive fearful-face task blocks, in the* ***anorexia nervosa (AN)*** *group:* ***(a)*** *F-values for block3 > block2 > block1 comparison,* ***(b)*** *T-values for block3 > block2 comparison,* ***(c)*** *T-values for block2 > block1 comparison,* ***(d)*** *p-values for block3 > block2 comparison,* ***(e)*** *p-values for block2 > block1 comparison,* ***(f)*** *effect sizes (Cohen's d) for block3 > block2 comparison,* ***(g)*** *effect sizes for block2 > block1 comparison. Those marked in red were statistically significant (p<0.05, Bonferroni corrected), while those in gray were not. The direction of the connections were from the regions mentioned in the rows to those in the columns, as indicated by the red arrow. T-value threshold corresponding to p<0.05 (Bonferroni corrected) is 3.17. Abbreviations: rACC = rostral anterior cingulate cortex; mPFC = medial prefrontal cortex; amyg = amygdala.*

F-value- BDD: block3 > block2 > block1

→	Amyg_L	Amyg_R	rACC_L	rACC_R	mPFC_L	mPFC_R
**Amyg_L**	0	0.51	0.22	0.12	0.13	0.37
**Amyg_R**	0.23	0	0.05	0.25	0.26	0.18
**rACC_L**	0.05	0.07	0	1.77	0.80	0.18
**rACC_R**	0.34	0.01	0.70	0	0.08	**4.36**
**mPFC_L**	0.42	0.97	1.04	0.08	0	0.73
**mPFC_R**	0.12	0.01	0.01	0.01	0.00	0

(a)

T-value- AN: block3 > block2

	Amyg_L	Amyg_R	rACC_L	rACC_R	mPFC_L	mPFC_R
**Amyg_L**	0	1.35	0.95	0.57	0.47	0.83
**Amyg_R**	0.63	0	0.76	0.89	1.12	0.38
**rACC_L**	0.43	1.01	0	3.22	2.22	0.56
**rACC_R**	0.81	0.11	0.83	0	0.36	**3.32**
**mPFC_L**	1.83	2.11	2.03	0.67	0	1.64
**mPFC_R**	0.54	-0.01	0.25	-0.31	0.09	0

(b)

T-value- AN: block2 > block1

	Amyg_L	Amyg_R	rACC_L	rACC_R	mPFC_L	mPFC_R
**Amyg_L**	0	1.16	0.65	0.64	0.82	1.31
**Amyg_R**	1.06	0	0.07	0.83	0.55	1.12
**rACC_L**	0.33	-0.66	0	1.74	0.87	0.96
**rACC_R**	1.21	0.26	1.95	0	0.62	**5.84**
**mPFC_L**	0.11	1.61	1.96	0.39	0	1.49
**mPFC_R**	0.89	0.44	-0.10	0.51	-0.26	0

(c)

P-value- AN: block3 > block2

	Amyg_L	Amyg_R	rACC_L	rACC_R	mPFC_L	mPFC_R
**Amyg_L**	0	0.18	0.34	0.57	0.64	0.41
**Amyg_R**	0.53	0	0.45	0.37	0.27	0.70
**rACC_L**	0.67	0.31	0	0.0014	0.03	0.57
**rACC_R**	0.42	0.91	0.41	0	0.72	**9.9×10^{-04}**
**mPFC_L**	0.07	0.04	0.04	0.50	0	0.10
**mPFC_R**	0.59	1.00	0.81	0.76	0.93	0

(d)

P-value- AN: block2 > block1

	Amyg_L	Amyg_R	rACC_L	rACC_R	mPFC_L	mPFC_R
**Amyg_L**	0	0.25	0.52	0.52	0.41	0.19
**Amyg_R**	0.29	0	0.94	0.41	0.58	0.26
**rACC_L**	0.74	0.51	0	0.08	0.38	0.34
**rACC_R**	0.23	0.79	0.05	0	0.54	**1.2×10^{-08}**
**mPFC_L**	0.91	0.11	0.05	0.70	0	0.14
**mPFC_R**	0.38	0.66	0.92	0.61	0.79	0

(e)

Effect size (Cohen's *d*): block3 vs. block2

	Amyg_L	Amyg_R	rACC_L	rACC_R	mPFC_L	mPFC_R
**Amyg_L**	0	0.14	0.10	0.06	0.05	0.09
**Amyg_R**	0.07	0	0.08	0.10	0.12	0.04
**rACC_L**	0.05	0.11	0	0.32	0.24	0.06
**rACC_R**	0.09	0.01	0.09	0	0.04	**0.36**
**mPFC_L**	0.20	0.23	0.22	0.07	0	0.18
**mPFC_R**	0.06	0.00	0.03	-0.03	0.01	0

(f)

Effect size (Cohen's *d*): block2 vs. block1

	Amyg_L	Amyg_R	rACC_L	rACC_R	mPFC_L	mPFC_R
**Amyg_L**	0	0.12	0.07	0.07	0.09	0.14
**Amyg_R**	0.11	0	0.01	0.09	0.06	0.12
**rACC_L**	0.04	-0.07	0	0.19	0.09	0.10
**rACC_R**	0.13	0.03	0.21	0	0.07	**0.62**
**mPFC_L**	0.01	0.17	0.21	0.04	0	0.16
**mPFC_R**	0.09	0.05	-0.01	0.05	-0.03	0

(g)

Table S5. *Statistics for the* ***between-group*** *differences in effective connectivity (averaged across all task blocks) between the* ***control*** *and the* ***BDD*** *groups:* ***(a)*** *T-values for Control > BDD comparison,* ***(b)*** *p-values for Control > BDD comparison,* ***(c)*** *effect sizes (Cohen's d) for Control > BDD comparison. Those marked in red were statistically significant (t-test, p<0.05, Bonferroni corrected), while those in gray were not. The direction of the connections were from the regions mentioned in the rows to those in the columns, as indicated by the red arrow. T-value threshold corresponding to p<0.05 (Bonferroni corrected) is 3.15. Abbreviations: rACC = rostral anterior cingulate cortex; mPFC = medial prefrontal cortex; amyg = amygdala.*

T-value: Control > BDD (all blocks combined)

→	Amyg_L	Amyg_R	rACC_L	rACC_R	mPFC_L	mPFC_R
**Amyg_L**	0	1.78	-1.58	-0.08	1.07	0.90
**Amyg_R**	0.64	0	0.11	-0.25	1.05	1.97
**rACC_L**	-1.24	0.83	0	-1.59	0.93	0.33
**rACC_R**	-0.92	-2.17	-0.25	0	-1.08	0.16
**mPFC_L**	-1.03	0.21	1.31	0.63	0	0.76
**mPFC_R**	-0.30	-0.56	-1.11	-0.38	-0.98	0

(a)

P-value: Control > BDD (all blocks combined)

→	Amyg_L	Amyg_R	rACC_L	rACC_R	mPFC_L	mPFC_R
**Amyg_L**	0	0.07	0.11	0.93	0.29	0.37
**Amyg_R**	0.52	0	0.91	0.80	0.29	0.05
**rACC_L**	0.21	0.41	0	0.11	0.35	0.74
**rACC_R**	0.36	0.03	0.80	0	0.28	0.87
**mPFC_L**	0.30	0.83	0.19	0.53	0	0.45
**mPFC_R**	0.77	0.57	0.27	0.70	0.33	0

(b)

Effect sizes: Control > BDD (all blocks combined)

→	Amyg_L	Amyg_R	rACC_L	rACC_R	mPFC_L	mPFC_R
Amyg_L	0	0.09	-0.08	0.00	0.06	0.05
Amyg_R	0.03	0	0.01	-0.01	0.06	0.10
rACC_L	-0.06	0.04	0	-0.08	0.05	0.02
rACC_R	-0.05	-0.11	-0.01	0	-0.06	0.01
mPFC_L	-0.06	0.01	0.07	0.03	0	0.04
mPFC_R	-0.02	-0.03	-0.06	-0.02	-0.05	0

(c)

Table S6. *Statistics for the* ***between-group*** *differences in effective connectivity (averaged across all task blocks) between the* ***control*** *and the* ***AN*** *groups:* ***(a)*** *T-values for Control > AN comparison,* ***(b)*** *p-values for Control > AN comparison,* ***(c)*** *effect sizes (Cohen's d) for Control > AN comparison. Those marked in red were statistically significant (t-test, p<0.05, Bonferroni corrected), while those in gray were not. The direction of the connections were from the regions mentioned in the rows to those in the columns, as indicated by the red arrow. T-value threshold corresponding to p<0.05 (Bonferroni corrected) is 3.15. Abbreviations: rACC = rostral anterior cingulate cortex; mPFC = medial prefrontal cortex; amyg = amygdala.*

T-statistic: Control > AN (all blocks combined)

→	Amyg_L	Amyg_R	rACC_L	rACC_R	mPFC_L	mPFC_R
Amyg_L	0	1.92	-0.47	1.83	1.07	1.62
Amyg_R	-0.91	0	**3.21**	2.06	0.87	1.68
rACC_L	0.15	-0.24	0	-1.22	0.79	1.22
rACC_R	-0.92	-1.02	0.40	0	-2.16	-0.63
mPFC_L	-0.38	**3.18**	**5.98**	0.31	0	0.28
mPFC_R	-1.18	0.49	0.30	-1.15	0.57	0

(a)

P-value: Control > AN (all blocks combined)

	Amyg_L	Amyg_R	rACC_L	rACC_R	mPFC_L	mPFC_R
Amyg_L	0	0.06	0.64	0.07	0.28	0.11
Amyg_R	0.36	0	**1.4×10^{-03}**	0.04	0.39	0.09
rACC_L	0.88	0.81	0	0.22	0.43	0.22
rACC_R	0.36	0.31	0.69	0	0.03	0.53
mPFC_L	0.71	**1.5×10^{-03}**	**2.8×10^{-09}**	0.75	0	0.78
mPFC_R	0.24	0.62	0.77	0.25	0.57	0

(b)

Effect sizes: Control > AN (all blocks combined)

	Amyg_L	Amyg_R	rACC_L	rACC_R	mPFC_L	mPFC_R
Amyg_L	0	0.11	-0.03	0.10	0.06	0.09
Amyg_R	-0.05	0	**0.18**	0.12	0.05	0.09
rACC_L	0.01	-0.01	0	-0.07	0.04	0.07
rACC_R	-0.05	-0.06	0.02	0	-0.12	-0.03
mPFC_L	-0.02	**0.18**	**0.34**	0.02	0	0.02
mPFC_R	-0.07	0.03	0.02	-0.06	0.03	0

(c)

SI-2.3. Change in Effective Connectivity Across Task Blocks: Connection-wise Observations

In this section, we take a closer look at each statistically significant connection identified in this work (as noted in **Tables S2** through **S6**). Specifically, with illustrations, we present and discus the progression of the effective connectivity within every group across every task block (both fearful face and scrambled face). This would enable us to better understand the evolution of effective connectivity over the task progression. We present summary statistics of effective connectivity values for each task block for the following connections (each figure compares all the three groups):

1. Connections originating from the left *mPFC*:

   – $mPFC_L$-to-$amygdala_L$ connection (**Fig.S1**).

   – $mPFC_L$-to-$amygdala_R$ connection (**Fig.S2**).

   – $mPFC_L$-to-$rACC_L$ connection (**Fig.S3**).

2. Connections originating from *rACC*:

   – $rACC_L$-to-$amygdala_L$ connection (**Fig.S4**).

   – $rACC_L$-to-$amygdala_R$ connection (**Fig.S5**).

   – $rACC_R$-to-$mPFC_R$ connection (**Fig.S6**).

3. Connections originating from the *amygdalae*:

   – $Amygdala_L$-to-$mPFC_L$ connection (**Fig.S7**).

   – $Amygdala_R$-to-$mPFC_L$ connection (**Fig.S8**).

   – $Amygdala_L$-to-$rACC_L$ connection (**Fig.S9**).

All the illustrations are presented next, followed by a thorough discussion on each of them.

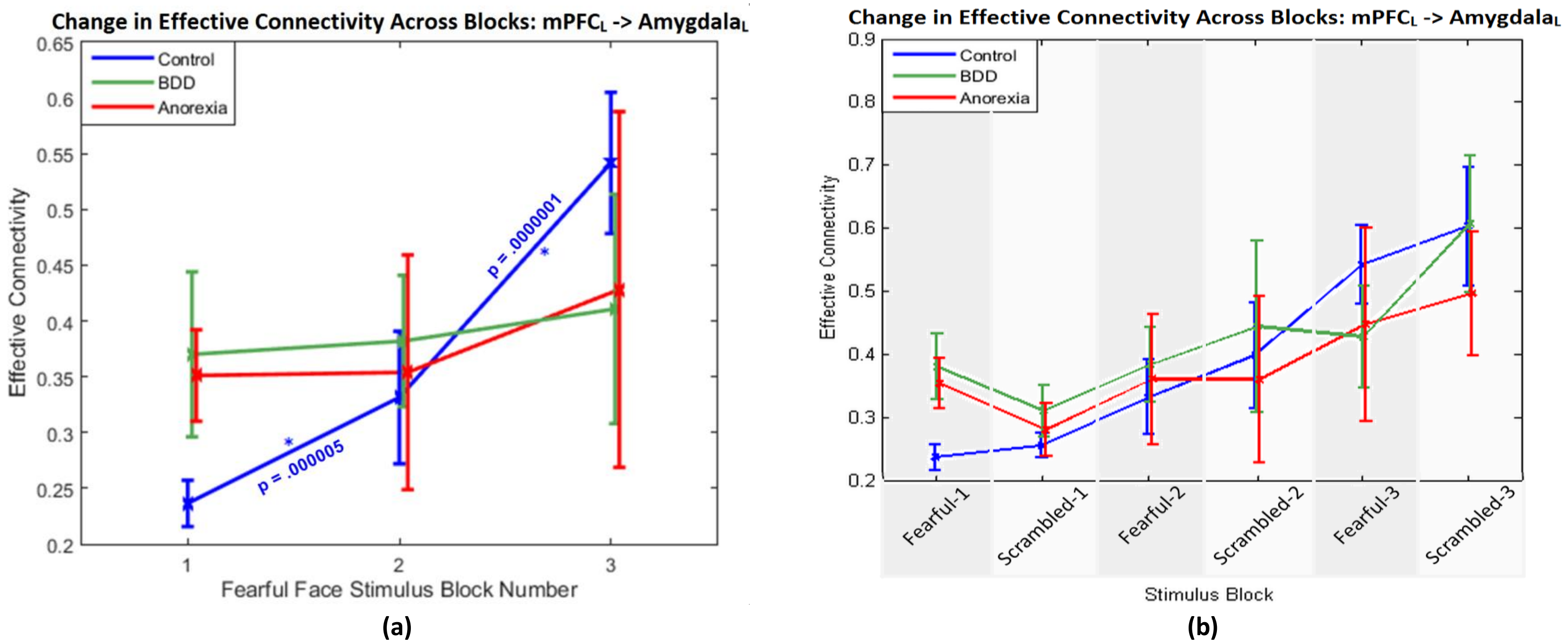

Fig.S1. *Within-group effective connectivity for the* ***left mPFC -to- left amygdala*** *connection:* ***(a)*** *shown across the three successive fearful-face task blocks, and* ***(b)*** *shown across all six task blocks (fearful-face-1 → scrambled-face-1 → fearful-face-2 → scrambled-face-2 → fearful-face-3 → scrambled-face-3). The p-values in (a) correspond to significant change in the within-group connectivity across the corresponding fearful-face blocks in the corresponding group (only those with marked p-values in (a) were statistically significant, p<0.05 Bonferroni corrected). P-values were not marked in (b) due to the enormous number of possible statistical comparisons. Abbreviations: mPFC = medial prefrontal cortex.*

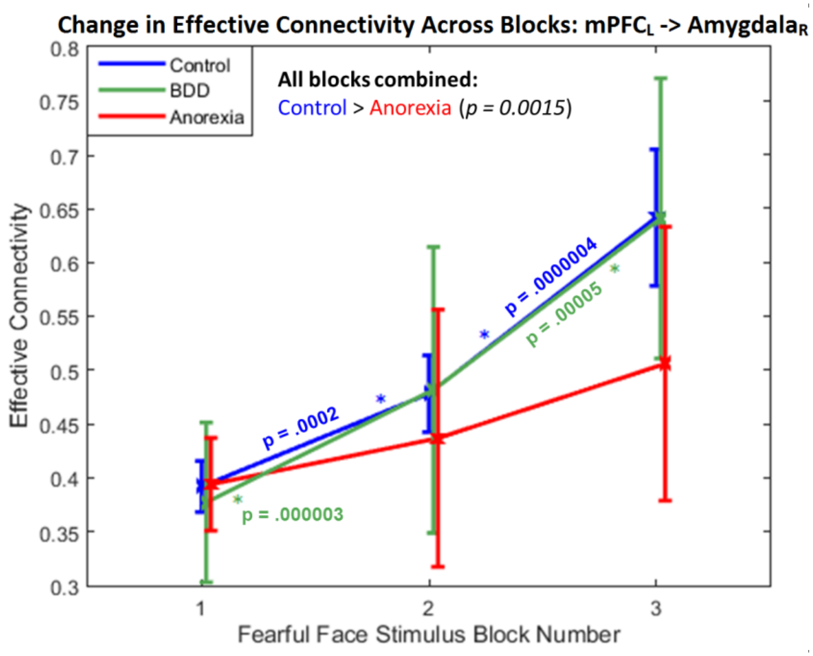

(a)

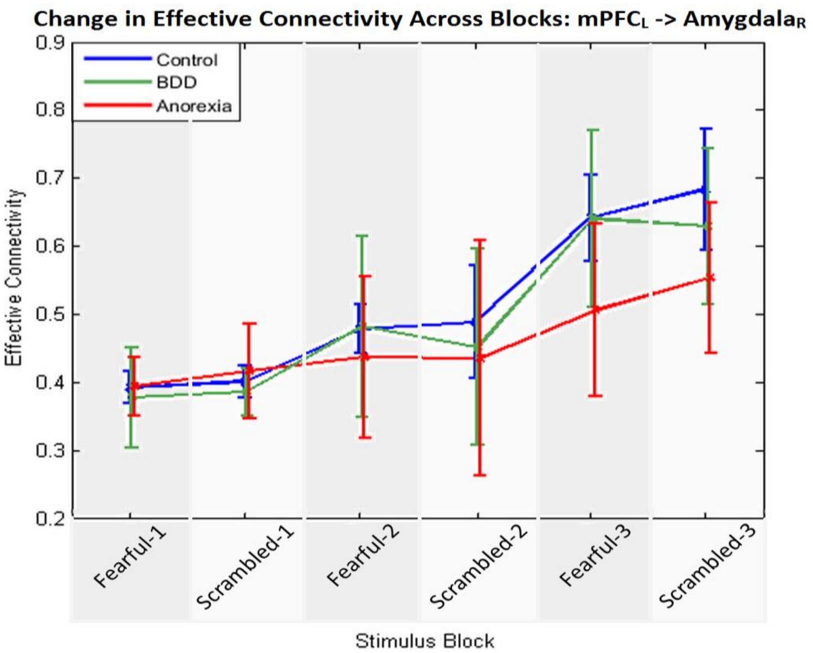

(b)

Fig.S2. *Within-group effective connectivity for the* ***left mPFC -to- right amygdala*** *connection:* ***(a)*** *shown across the three successive fearful-face task blocks, and* ***(b)*** *shown across all six task blocks (fearful-face-1 → scrambled-face-1 → fearful-face-2 → scrambled-face-2 → fearful-face-3 → scrambled-face-3). The p-values on the lines in (a) correspond to significant change in the within-group connectivity across the corresponding fearful-face blocks in the corresponding group (only those with marked p-values in (a) were statistically significant, p<0.05 Bonferroni corrected); while the p-value shown for the "Control > Anorexia" comparison corresponds to the between-group difference in connectivity across all blocks. P-values were not marked in (b) due to the enormous number of possible statistical comparisons. Abbreviations: mPFC = medial prefrontal cortex.*

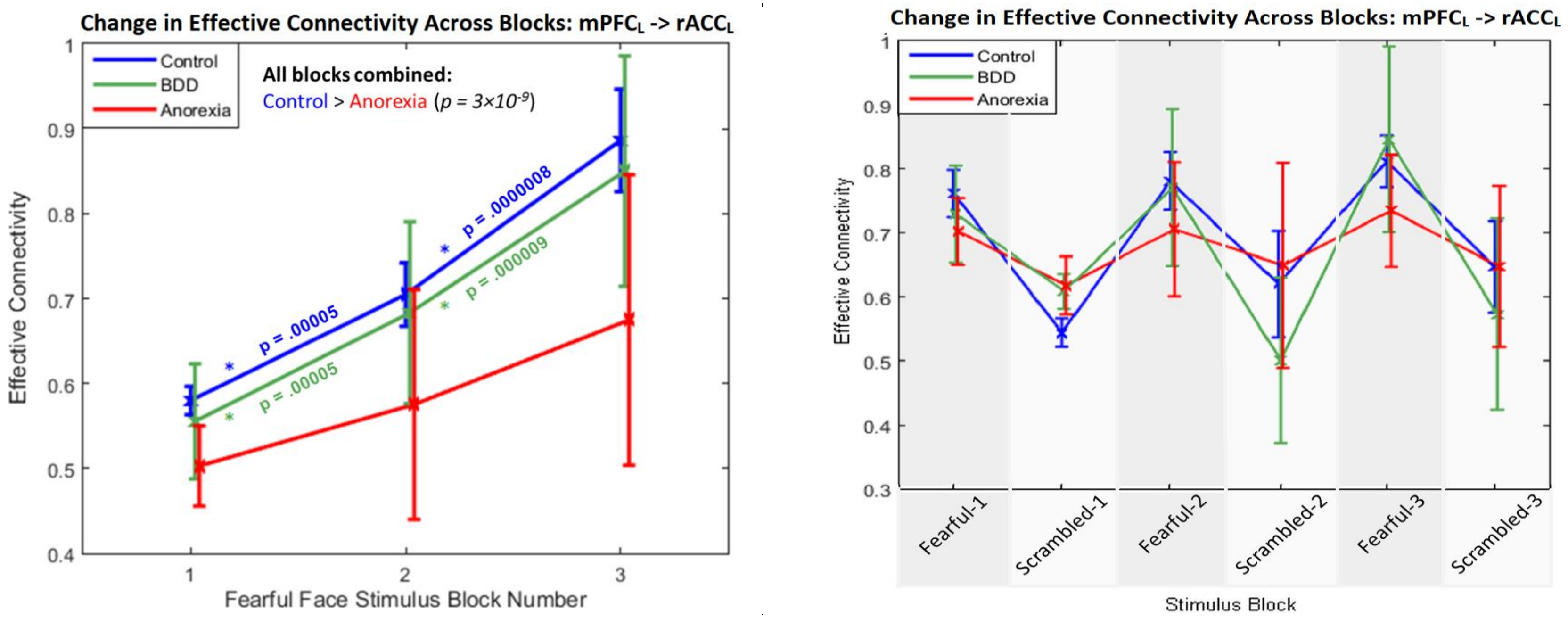

(a) (b)

Fig.S3. *Within-group effective connectivity for the* **left mPFC -to- left rACC** *connection:* **(a)** *shown across the three successive fearful-face task blocks, and* **(b)** *shown across all six task blocks (fearful-face-1 → scrambled-face-1 → fearful-face-2 → scrambled-face-2 → fearful-face-3 → scrambled-face-3). The p-values on the lines in (a) correspond to significant change in the within-group connectivity across the corresponding fearful-face blocks in the corresponding group (only those with marked p-values in (a) were statistically significant, p<0.05 Bonferroni corrected); while the p-value shown for the "Control > Anorexia" comparison corresponds to the between-group difference in connectivity across all blocks. P-values were not marked in (b) due to the enormous number of possible statistical comparisons. Abbreviations: rACC=rostral anterior cingulate cortex; mPFC=medial prefrontal cortex.*

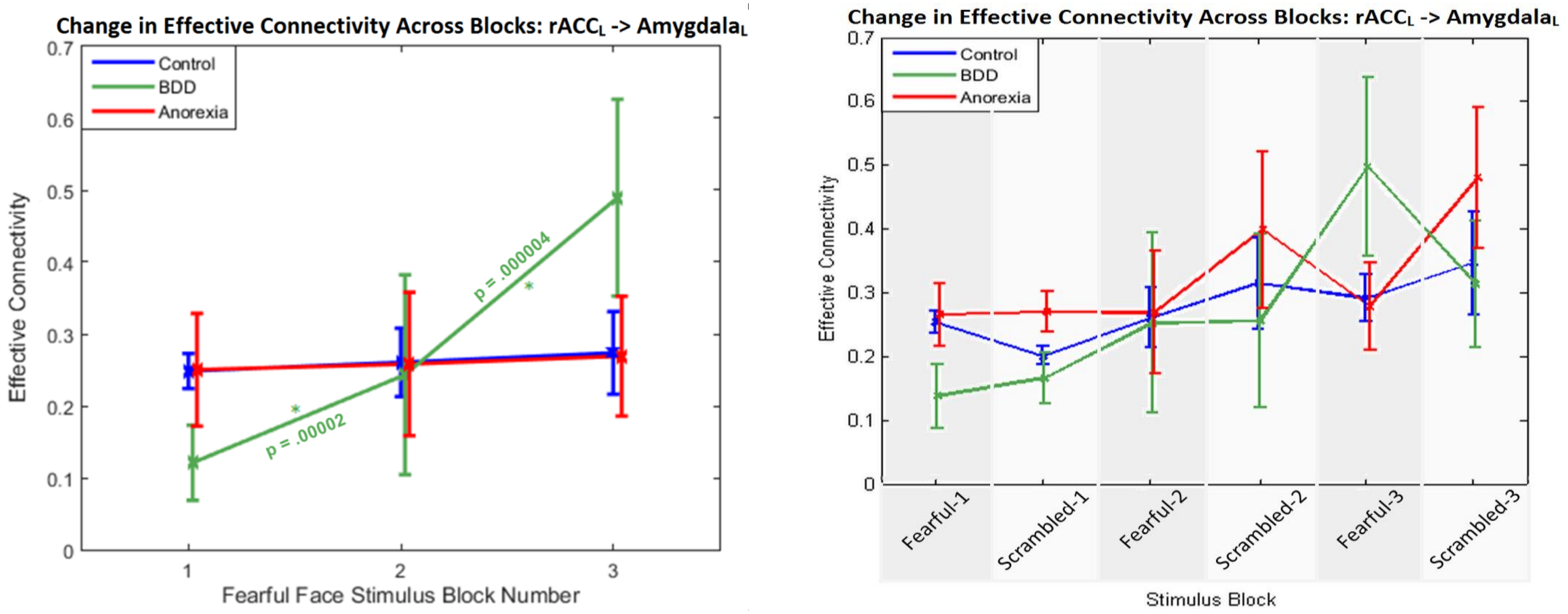

(a) **(b)**

Fig.S4. *Within-group effective connectivity for the* ***left rACC -to- left amygdala*** *connection:* ***(a)*** *shown across the three successive fearful-face task blocks, and* ***(b)*** *shown across all six task blocks (fearful-face-1 → scrambled-face-1 → fearful-face-2 → scrambled-face-2 → fearful-face-3 → scrambled-face-3). The p-values in (a) correspond to significant change in the within-group connectivity across the corresponding fearful-face blocks in the corresponding group (only those with marked p-values in (a) were statistically significant, $p<0.05$ Bonferroni corrected). P-values were not marked in (b) due to the enormous number of possible statistical comparisons. Abbreviations: rACC = rostral anterior cingulate cortex.*

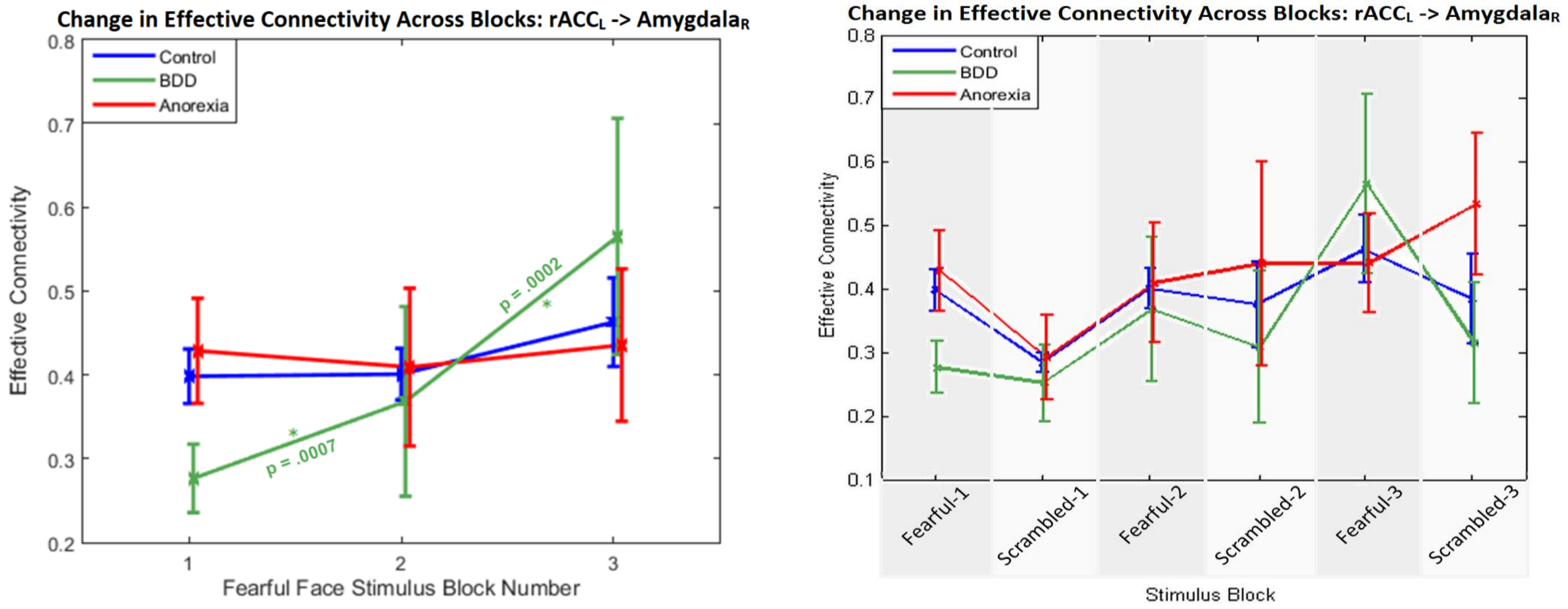

(a) **(b)**

Fig.S5. *Within-group effective connectivity for the* ***left rACC -to- right amygdala*** *connection:* ***(a)*** *shown across the three successive fearful-face task blocks, and* ***(b)*** *shown across all six task blocks (fearful-face-1 → scrambled-face-1 → fearful-face-2 → scrambled-face-2 → fearful-face-3 → scrambled-face-3). The p-values in (a) correspond to significant change in the within-group connectivity across the corresponding fearful-face blocks in the corresponding group (only those with marked p-values in (a) were statistically significant, p<0.05 Bonferroni corrected). P-values were not marked in (b) due to the enormous number of possible statistical comparisons. Abbreviations: rACC = rostral anterior cingulate cortex.*

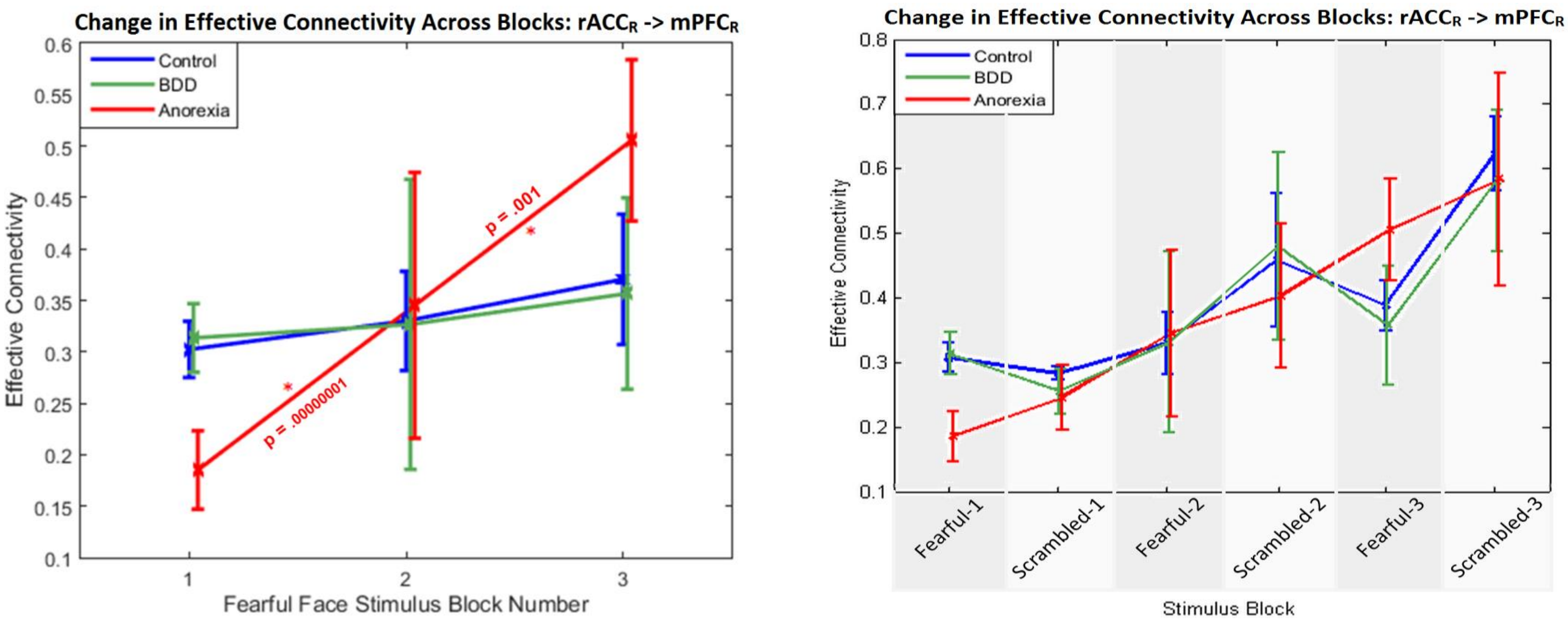

(a) **(b)**

Fig.S6. *Within-group effective connectivity for the* ***right rACC -to- right mPFC*** *connection:* ***(a)*** *shown across the three successive fearful-face task blocks, and* ***(b)*** *shown across all six task blocks (fearful-face-1 → scrambled-face-1 → fearful-face-2 → scrambled-face-2 → fearful-face-3 → scrambled-face-3). The p-values in (a) correspond to significant change in the within-group connectivity across the corresponding fearful-face blocks in the corresponding group (only those with marked p-values in (a) were statistically significant, p<0.05 Bonferroni corrected). P-values were not marked in (b) due to the enormous number of possible statistical comparisons. Abbreviations: rACC = rostral anterior cingulate cortex; mPFC = medial prefrontal cortex.*

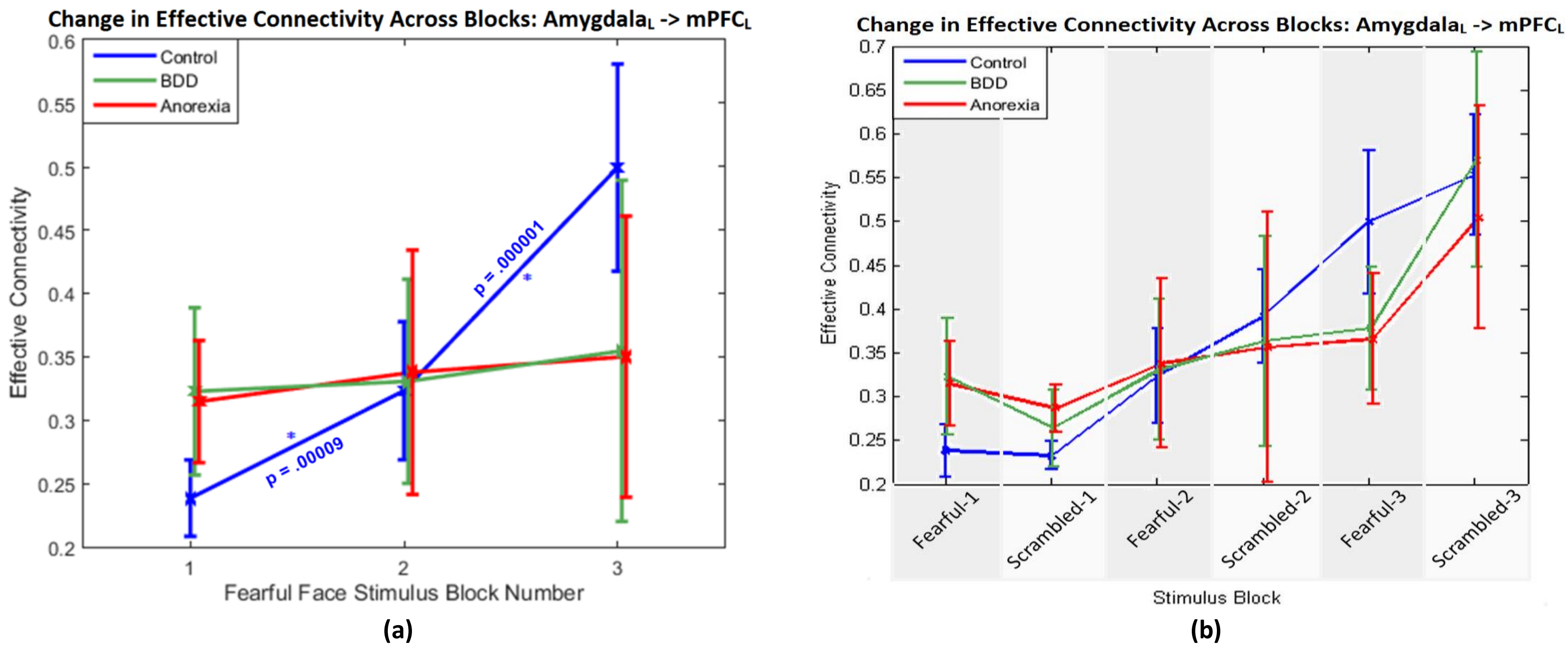

Fig.S7. *Within-group effective connectivity for the* ***left amygdala -to- left mPFC*** *connection:* ***(a)*** *shown across the three successive fearful-face task blocks, and* ***(b)*** *shown across all six task blocks (fearful-face-1 → scrambled-face-1 → fearful-face-2 → scrambled-face-2 → fearful-face-3 → scrambled-face-3). The p-values in (a) correspond to significant change in the within-group connectivity across the corresponding fearful-face blocks in the corresponding group (only those with marked p-values in (a) were statistically significant, p<0.05 Bonferroni corrected). P-values were not marked in (b) due to the enormous number of possible statistical comparisons. Abbreviations: mPFC = medial prefrontal cortex.*

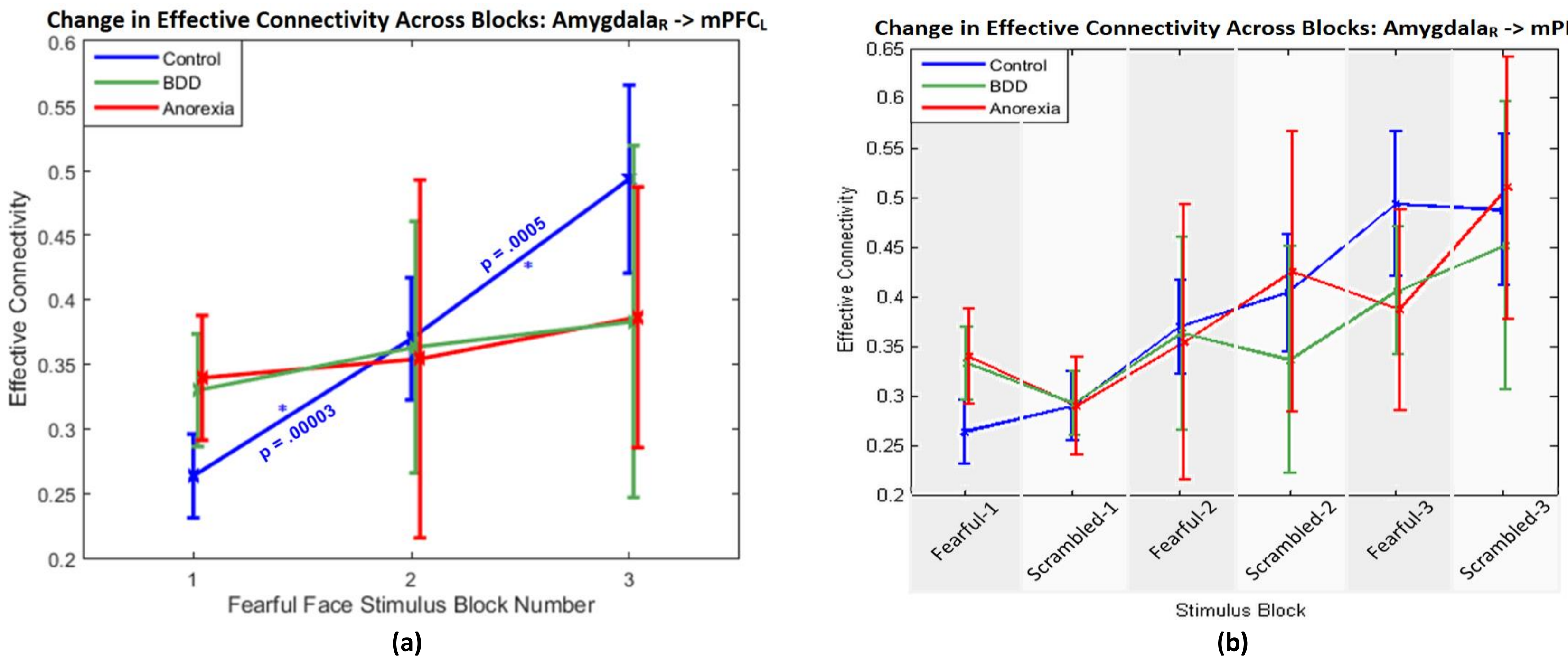

Fig.S8. *Within-group effective connectivity for the* **right amygdala -to- left mPFC** *connection:* ***(a)*** *shown across the three successive fearful-face task blocks, and* ***(b)*** *shown across all six task blocks (fearful-face-1 → scrambled-face-1 → fearful-face-2 → scrambled-face-2 → fearful-face-3 → scrambled-face-3). The p-values in (a) correspond to significant change in the within-group connectivity across the corresponding fearful-face blocks in the corresponding group (only those with marked p-values in (a) were statistically significant, p<0.05 Bonferroni corrected). P-values were not marked in (b) due to the enormous number of possible statistical comparisons. Abbreviations: mPFC = medial prefrontal cortex.*

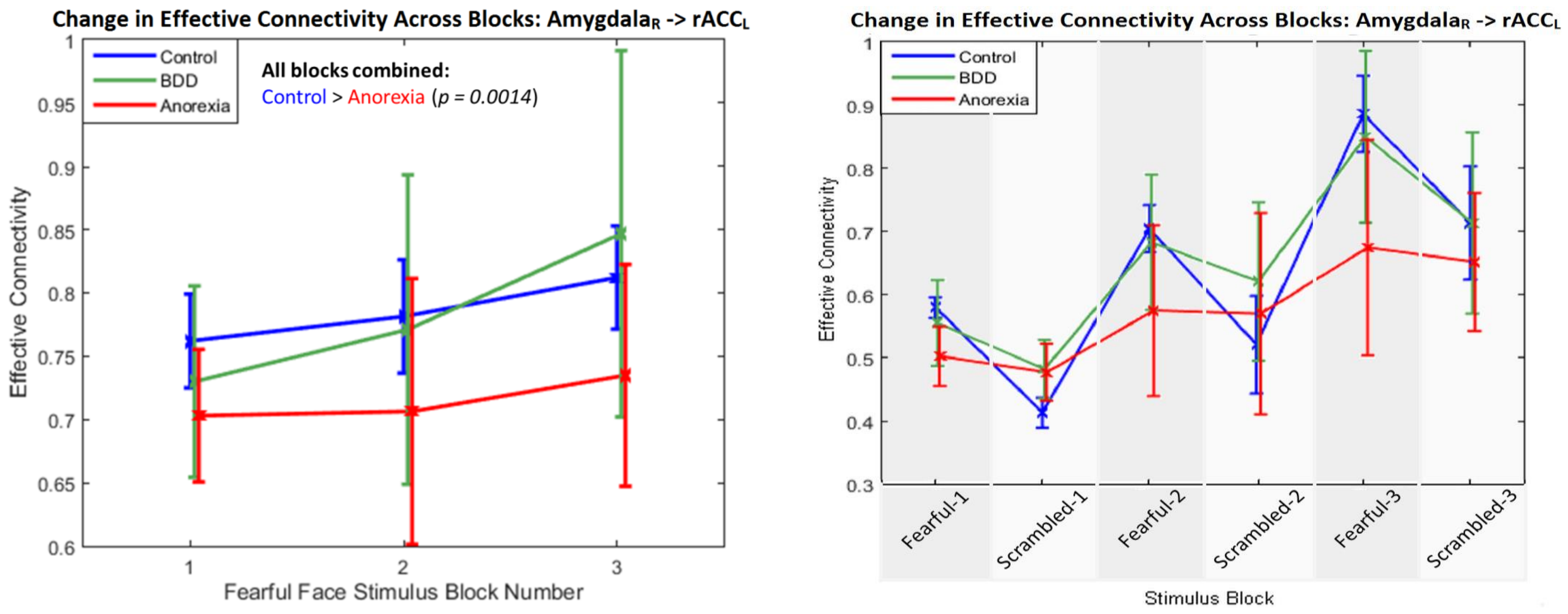

(a) (b)

Fig.S9. *Within-group effective connectivity for the* ***right amygdala -to- left rACC*** *connection:* ***(a)*** *shown across the three successive fearful-face task blocks, and* ***(b)*** *shown across all six task blocks (fearful-face-1 → scrambled-face-1 → fearful-face-2 → scrambled-face-2 → fearful-face-3 → scrambled-face-3). The p-value shown for the "Control > Anorexia" comparison corresponds to the between-group difference in connectivity across all blocks. P-values were not marked in (b) due to the enormous number of possible statistical comparisons. Abbreviations: rACC = rostral anterior cingulate cortex.*

Effective connectivity across the three fearful-face blocks were presented to provide insight into the change in connectivity over the course of repeated exposure to fearful faces; while the figures illustrating the change in effective connectivity across all six task blocks (including scrambled faces) were presented to provide insight into the transition from fearful-face to scrambled-face stimuli and from scrambled-face to fearful-face stimuli over the task progression.

Here are the observations that can be drawn from **Figures S1** through **S9**:

1. The *left mPFC -to- left amygdala* connectivity (**Fig.S1**): With repeated fearful faces, we observed enhanced engagement in the control group (*block3 vs block2 vs block1:* $F_{3,108}=6.2$, $P=5.4\times10^{-09}$; *block3 vs block2:* $T=5.34$, $P=1.4\times10^{-07}$; *block2 vs block1:* $T=4.61$, $P=5.2\times10^{-06}$), but not in the BDD or AN groups ($p>0.05$). In addition, the initial connectivity in the control group (i.e. the first block) was lower than that in the BDD ($T=1.89$, $P=2.9\times10^{-02}$) or AN ($T=4.07$, $P=2.7\times10^{-05}$) groups. Observations regarding this connection seem similar to the observations regarding the *left mPFC -to- right amygdala* connectivity (**Fig.S2**, which has also been discussed in detail in the main text).

2. The *left mPFC -to- right amygdala* connectivity (**Fig.S2**): This connection has been discussed in detail in the main text. Here we note that effective connectivity progression across all six task blocks shows a trend similar to that observed in the fearful-face blocks alone, with enhanced connectivity across task blocks observed in the control and BDD groups but not in the AN group.

3. The *left mPFC -to- left rACC* connectivity (**Fig.S3**): With repeated fearful faces, the control and BDD groups, but not the AN group, showed significantly enhanced engagement of this connection (*Control, block3 vs block2 vs block1:* $F_{3,108}=5.76$, $P=9.5\times10^{-08}$; *block3 vs*

block2: T=4.99, $P=8.2\times10^{-07}$*; block2 vs block1: T=4.07,* $P=5.3\times10^{-05}$) (*BDD, block3 vs block2 vs block1:* $F_{3,108}=4.94$, $P=2.1\times10^{-07}$*; block3 vs block2: T=4.48,* $P=9.5\times10^{-06}$*; block2 vs block1: T=4.09,* $P=5.2\times10^{-05}$). Additionally, the AN group exhibited significantly lower average connectivity than the control (*T=5.98,* $P=2.8\times10^{-09}$) group (between-group comparison). All between-group comparisons in this study were controlled for head motion, age, sex and education ($p<0.05$, Bonferroni corrected).

In the context of this connection, we next discus two contrasting trends of connectivity progression across the six task blocks (alternating fearful and scrambled faces) observed among the nine connections identified in this work. While the bilateral bidirectional mPFC-amygdala connectivity (**Figs. S1, S2, S7** and **S8**) appeared to exhibit a trend of monotonic increase across all six task blocks, most connectivities involving the rACC exhibited a pattern of increased connectivity during fearful faces and decreased connectivity during scrambled faces among all the groups (all the within-group differences in connectivity between successive blocks were statistically significant in all the groups for the *left mPFC -to- left rACC* connectivity, $p<0.01$. This trend was also observed in another connection involving the rACC, the amygdala-to-rACC connectivity [**Fig.S9**]). Among the identified connections in this work, we thus observe a clear dichotomy, as described next:

(i) *The monotonically increasing response*: The behavioral response of fear inhibition in reaction to repeated fearful faces (like in our task design) tends to be monotonic in nature (Gross, 2014); in corroboration, the connections associated with the mPFC also exhibited a similar monotonically increasing trend across all six task blocks, hinting that we could expect mPFC connectivity to have significant mapping with subjective emotional experiences. In agreement with this, we also found an association between reported degree of fear and the

mPFC-to-amygdala connectivity during fearful-face blocks (r^2*=0.10, P=0.0016, 95%-CI=[0.02,0.24]*), as reported in the main text.

(ii) *The "saw-tooth" response (alternating increase and decrease in connectivity across task blocks)*: Most connections associated with the rACC exhibited such a response. One possible explanation for such a response (a response that is not similar to the 'monotonic' nature of behavioral expressions) is that the rACC is inherently more reactive to the emotional content (thereby more responsive during the fearful-face blocks), whereas the connections associated with the mPFC reflect more on evaluative coding of the face (Gross, 2014). The reason behind consistently lower mPFC-to-rACC connectivity during viewing of scrambled faces however remains unclear; future studies could develop and test hypothesis on the role of this connectivity during fearful and neutral face processing.

4. The *left rACC -to- left amygdala* connectivity (**Fig.S4**): In the main text we discussed that this connection often emerges in response to impaired prefrontal-amygdala connectivity (Kujawa, et al., 2016), and that the increased engagement of this connection in the BDD group (but not in the control or AN groups) could be an attempt by the brain as an alternative strategy to compensate for impaired amygdala-to-mPFC connectivity. It is also notable that its increased engagement is observed only in the fearful-face blocks in BDD (nearly a flat response in the scrambled-face blocks, $P>0.05$), possibly because the need to compensate for an impaired amygdala-to-mPFC connectivity arises only when presented with emotionally provocative stimuli.

5. The *left rACC -to- right amygdala* connectivity (**Fig.S5**): The trend is largely similar to the *left rACC -to- left amygdala* connectivity (**Fig.S4**); similar inferences can be drawn.

6. The *right rACC -to- right mPFC* connectivity (**Fig.S6**): This connection exhibited nearly a flat response in the control and BDD groups during successive fearful-face blocks (*P>0.5*). The control group, however, also exhibited trends of enhanced engagement of this connectivity during successive viewing of scrambled faces (*block3 vs block2 (trend): T=1.42, P=0.07; block2 vs block1 (significant): T=2.18,* $P=1.5\times10^{-02}$) (no trend or significance in BDD, *P>0.25*). In the AN group, monotonically increasing engagement was observed across all blocks, that is, during fearful-face as well as scrambled-face blocks (*fearful-1 vs scrambled-1: T=1.68, P=0.04; scrambled-1 vs fearful-2: T=3.29,* $P=5.5\times10^{-4}$*; fearful-2 vs scrambled-2 (not significant): T=0.55, P=0.29; scrambled-2 vs fearful-3: T=1.81, P=0.03; fearful-3 vs scrambled-3 (not significant): T=0.41, P=0.34*) (*comparison of fearful-face blocks only, block3 vs block2: T=3.32,* $P=9.9\times10^{-4}$*; block2 vs block1: T=5.84,* $P=1.2\times10^{-8}$). This connection, *right rACC -to- right mPFC,* is known to be elevated during situations of conflict (Nakao, et al., 2010), particularly a conflict in behavior selection. Hence, its monotonic elevated engagement in the AN group might relate to increasing conflict that could arise when attempting to choose an adaptive fear response in the face of repeated, impaired ability to do so. In other words, this connectivity profile might signify their efforts to compensate for their inability in responding adequately and conventionally to repeated fearful faces. In the control and BDD groups, it remains unclear as to why elevated conflict processing might manifest during viewing of repeated scrambled faces; though one possible explanation is that the impact of elevated fear arising from viewing of fearful faces continues after the termination of the fearful-face stimuli (Marek, Strobel, Bredy, & Sah, 2013), that is, fear response persists even after the fearful-face block, hence the state of

elevated fear coupled with the viewing of emotionally neutral (scrambled) faces creates a scenario of behavioral conflict.

7. The *left amygdala -to- left mPFC* connectivity (**Fig.S7**): This connectivity, which corresponds to the amygdala-to-mPFC feed-forward signaling, was found to exhibit a profile of significantly elevated engagement in the control group (*block3 vs block2 vs block1:* $F_{3,108}=5.3$, $P=1.8\times10^{-9}$; *block3 vs block2:* $T=4.96$, $P=9.5\times10^{-7}$; *block2 vs block1:* $T=3.95$, $P=9.1\times10^{-5}$), but not in the BDD or AN groups ($P>0.3$), during successive viewing of fearful faces. A similar profile was observed with the *right amygdala -to- left mPFC* connectivity (**Fig.S8**), and this connectivity has been discussed in detail in the main text.

8. The *right amygdala -to- left mPFC* connectivity (**Fig.S8**): The trend is largely similar to the *left amygdala -to- left mPFC* connectivity (**Fig.S7**); similar inferences can be drawn.

9. The *right amygdala -to- left rACC* connectivity (**Fig.S9**): This connectivity did not exhibit increasing engagement across successive fearful-face blocks in any group ($P>0.1$), but was significantly lower in magnitude in the AN group (averaged across all blocks) compared to the control ($T=3.21$, $P=1.4\times10^{-3}$) and BDD ($T=2.55$, $P=1.1\times10^{-2}$) groups. This connection has been previously reported as being involved during incidental perception (de Marco, de Bonis, Vrignaud, Henry-Feugeas, & Peretti, 2006) (for example viewing and rating faces); hence, its lower engagement in the AN group might provide preliminary evidence that the AN group might view/rate faces in general differently from HC and BDD groups. In addition to this, we also observed the trend of higher engagement during fearful-faces and lower engagement during scrambled-faces (all the within-group differences in connectivity between

successive blocks were statistically significant in all the groups, $p<0.03$), as observed in other connections involving the rACC (**Figs. S3** through **S6**).

SI-2.4. Discussion on associations between connectivity and behavior

We observed significant association between subjective fear ratings (SFR) and the mPFC-to-amygdala connectivity when participants from all the groups were taken together. However, when the groups were considered individually, a significant association was observed in the control ($r^2=0.22$, $P=0.0032$, *95%-CI=[0.17,0.69]*) and AN ($r^2=0.32$, $P=0.0035$, *95%-CI=[0.21,0.78]*) groups, but not in the BDD group ($r^2=0.0001$, $P=0.9560$). The association in the control and AN groups implies that this connection is a consequence of (or, alternatively, contributes to) the subjective experience of fear, with an increase in this connectivity corresponding to an increase in subjective fear, implying enhanced engagement of this connectivity with elevated fear response. By contrast, the absence of this association in BDD could be due to the limbic hypo-responsiveness observed in this disorder (Feusner, et al., 2010), wherein an absence of limbic hyperactivity can be observed when participants view their own faces, despite high subjective rating of emotional discomfort. This dissociation between connectivity and subjective emotional experience might be the driving factor behind the absence of the association. A similar trait is observed even in obsessive-compulsive disorder (OCD), a condition closely related to BDD, wherein anxious arousal upon stimulus exposure is seen without corresponding amygdala hyperactivity in most (but not all) studies (Rotge, et al., 2008).

On a side note, a couple of negative observations deserve mentioning. First, both SFR and the HAMA scores were found to be associated with the mPFC-to-amygdala connectivity in AN, which raises the question if these measures of fear and anxiety are associated themselves. We

found that SFR and HAMA were not associated with each other in either the BDD (*r^2=0.08, P=0.1760*) or the AN (*r^2=0.01, P=0.6690*) groups, which might reflect the notion that fear and anxiety are different constructs (Gross, 2014). (In the control group HAMA scores were overall too low for meaningful analyses.) Second, we found no significant associations between lowest lifetime BMI or duration of illness with any of the connections in the AN group. Noting that we recruited weight-restored AN participants in this study, this observation implies that the history of the illness in these participants – in terms of how long they had been ill, or how low their BMI had been – had no significant impact on the connectivity profile observed in this group.

Supplemental References

de Marco, G., de Bonis, M., Vrignaud, P., Henry-Feugeas, M., & Peretti, I. (2006). Changes in effective connectivity during incidental and intentional perception of fearful faces. *Neuroimage, 30*(3), 1030-7.

Enander, J., Evanov, V., Andersson, E., Mataix-Cols, D., Ljótsson, B., & Rück, C. (2014). Therapist-guided, internet-based cognitive-behavioural therapy for body dysmorphic disorder (BDD-NET): a feasibility study. *BMJ Open, 4*(9), e005923.

Feusner, J., Moody, T., Hembacher, E., Townsend, J., McKinley, M., Moller, H., & Bookheimer, S. (2010). Abnormalities of visual processing and frontostriatal systems in body dysmorphic disorder. *Arch Gen Psychiatry, 67*, 197-205.

Friston, K., Rotshtein, P., Geng, J., Sterzer, P., & Henson, R. (2006). A critique of functional localisers. *NeuroImage, 30*, 1077-1087.

Gross, J. (2014). *Handbook of Emotion Regulation.* Ney York: The Guilford Press.

Kujawa, A., Wu, M., Klumpp, H., Pine, D., Swain, J., Fitzgerald, K., . . . Phan, K. (2016). Altered Development of Amygdala-Anterior Cingulate Cortex Connectivity in Anxious Youth and Young Adults. *Biol Psychiatry Cogn Neurosci Neuroimaging., 1*(4), 345-352.

Labudda, K., Mertens, M., Steinkroeger, C., Bien, C., & Woermann, F. (2014). Lesion side matters — An fMRI study on the association between neural correlates of watching dynamic fearful faces and their evaluation in patients with temporal lobe epilepsy. *Epilepsy & Behavior, 31*, 321-328.

Marek, R., Strobel, C., Bredy, T., & Sah, P. (2013). The amygdala and medial prefrontal cortex: partners in the fear circuit. *J Physiol, 591*, 2381-2391.

Nakao, T., Osumi, T., Ohira, H., Kasuya, Y., Shinoda, J., Yamada, J., & Northoff, G. (2010). Medial prefrontal cortex-dorsal anterior cingulate cortex connectivity during behavior selection without an objective correct answer. *Neurosci Lett., 482*(3), 220-4.

Rotge, J.-Y., Guehl, D., Dilharreguy, B., Cuny, E., Tignol, J., Bioulac, B., . . . Aouizerate, B. (2008). Provocation of obsessive–compulsive symptoms: a quantitative voxel-based meta-analysis of functional neuroimaging studies. *J Psychiatry Neurosci, 33*, 405-412.

Veale, D., Anson, M., Miles, S., Pieta, M., Costa, A., & Ellison, N. (2014). Efficacy of cognitive behaviour therapy versus anxiety management for body dysmorphic disorder: a randomized controlled trial. *Psychother Psychosom, 83*, 341-353.
